# A New Titanosaurian Sauropod from the Hekou Group (Lower Cretaceous) of the Lanzhou-Minhe Basin, Gansu Province, China

**DOI:** 10.1371/journal.pone.0085979

**Published:** 2014-01-29

**Authors:** Li-Guo Li, Da-Qing Li, Hai-Lu You, Peter Dodson

**Affiliations:** 1 School of Earth Sciences and Resources, China University of Geosciences (Beijing), Beijing, China; 2 School of Veterinary Medicine, University of Pennsylvania, Philadelphia, Pennsylvania, United States of America; 3 Gansu Geological Museum, Lanzhou, China; 4 Key Laboratory of Vertebrate Evolution and Human Origins of Chinese Academy of Sciences, Institute of Vertebrate Paleontology and Paleoanthropology, Chinese Academy of Sciences, Beijing, P. R. China; University of Birmingham, United Kingdom

## Abstract

Increased excavation of dinosaurs from China over the last two decades has enriched the record of Asian titanosauriform sauropods. However, the relationships of these sauropods remain contentious, and hinges on a few well-preserved taxa, such as *Euhelopus zdanskyi*. Here we describe a new sauropod, *Yongjinglong datangi* gen. nov. et sp. nov., from the Lower Cretaceous Hekou Group in the Lanzhou Basin of Gansu Province, northwestern China. *Yongjinglong datangi* is characterized by the following unique combination of characters, including seven autapomorphies: long-crowned, spoon-shaped premaxillary tooth; axially elongate parapophyses on the cervical vertebra; very deep lateral pneumatic foramina on the lateral surfaces of the cervical and cranial dorsal vertebral centra; low, unbifurcated neural spine fused with the postzygapophyses to form a cranially-pointing, triangular plate in a middle dorsal vertebra; an “XI”-shaped configuration of the laminae on the arches of the middle dorsal vertebrae; a very long scapular blade with straight cranial and caudal edges; and a tall, deep groove on the lateral surface of the distal shaft of the radius. The new specimen shares several features with other sauropods: a pronounced *M. triceps longus* tubercle on the scapula and ventrolaterally elongated parapophyses in its cervical vertebra as in Euhelopodidae. Based on phylogenetic analyses *Yongjinglong datangi* is highly derived within Titanosauria, which suggests either a remarkable convergence with more basal titanosauriform sauropods in the Early Cretaceous or a retention of plesiomorphic features that were lost in other titanosaurians. The morphology and remarkable length of the scapulocoracoid reveal an unusual relationship between the shoulder and the middle trunk: the scapulocoracoid spans over half of the length of the trunk. The medial, notch-shaped coracoid foramen and the partially fused scapulocoracoid synostosis suggest that the specimen is a subadult individual. This specimen sheds new light on the diversity of Early Cretaceous Titanosauriformes in China.

## Introduction

**Figure 1:**
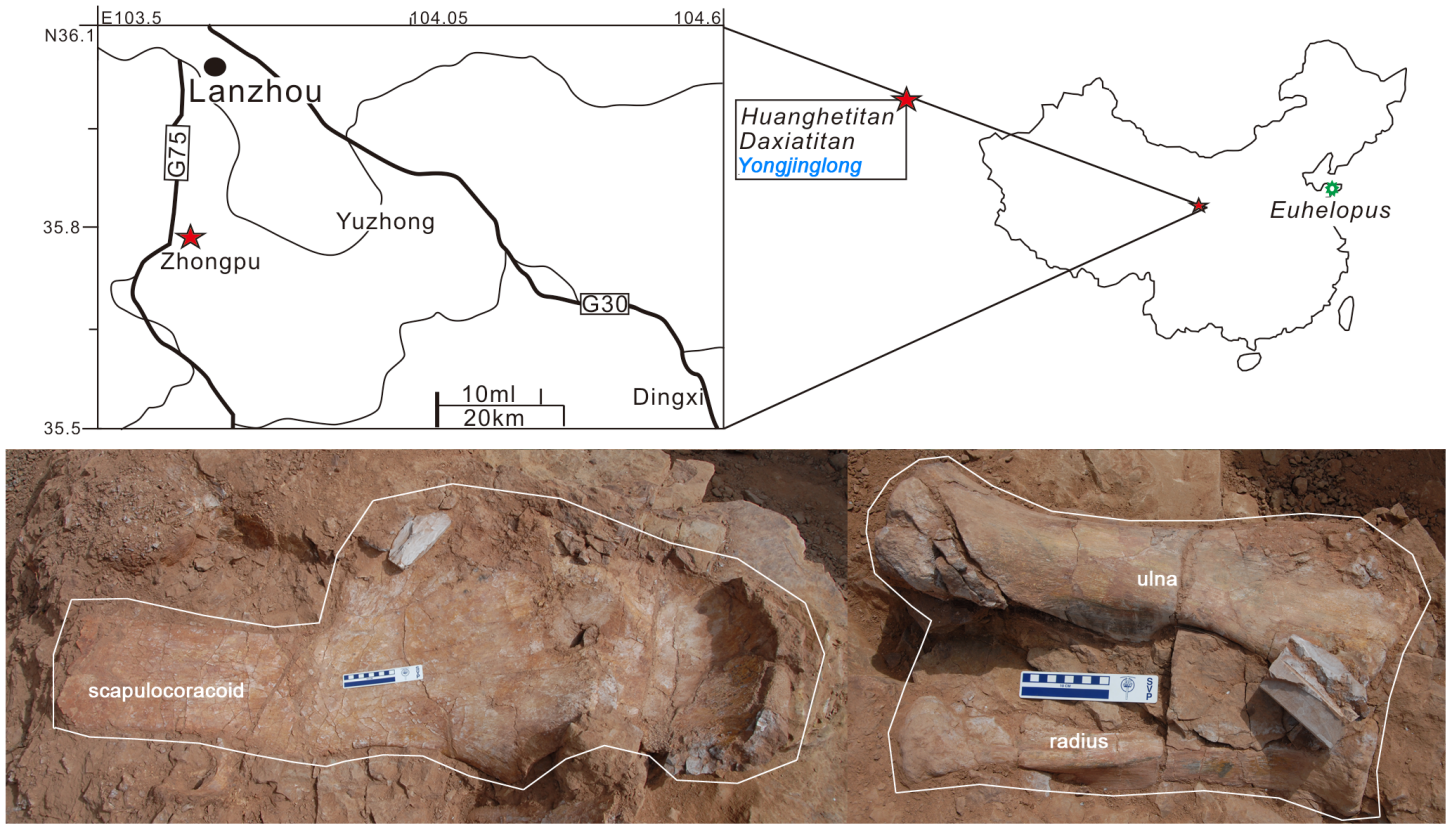
Fossil locations and quarry map of *Yongjinglong datangi*. In the upper picture the red star presents the fossil locations of *Yongjinglong, Daxiatitan* and *Huanghetitan* and also the green star shows the excavation location of *Euhelopus*. In the lower picture the exposed fossils are marked.

The earliest reported Chinese sauropod dinosaur, *Euhelopus zdanskyi* (originally *Helopus zdanskyi*) from Shandong Province, eastern China, was described in 1929 by the Swedish paleontologist Carl Wiman [1]; additional material was described in 1935 by C.-C. Young [2] (Fig.1). During the subsequent half century, the most famous sauropods found in China include *Omeisaurus* [3], [4], *Shunosaurus* [5], [6] and *Mamenchisaurus* [7]–[11], all of which come from the Middle to Upper Jurassic strata of the Sichuan Basin, southwestern China [6], [10]. More recently, Cretaceous sauropods have been excavated in several areas of China, especially in Gansu Province, northwestern China. These include four Early Cretaceous taxa: *Gobititan shenzhouensis* [12], *Huanghetitan liujiaxiaensis* [13], *Daxiatitan binglingi* [14], and *Qiaowanlong kangxii* [15]. Most of these sauropods were originally described as basal members of Titanosauriformes. In the latest studies, however, *Qiaowanlong* was recovered as a derived member of Somphospondyli, rather than in Brachiosauridae [16]–[18], and has been assigned to Euhelopodidae, a newly defined clade of Somphospondyli comprising *Euhelopus* and possibly several other contemporaneous East Asian sauropods, including *Daxiatitan* [19] (though see [18] for a partly contrasting view). Here we describe another new sauropod, *Yongjinglong datangi* gen. nov. et sp. nov., from the Lower Cretaceous Hekou Group in the Lanzhou-Minhe Basin, Gansu Province, the same strata that yielded *Huanghetitan liujiaxiaensis* and *Daxiatitan.* The new taxon is based on a specimen that comprises three isolated teeth, eight vertebrae, the left scapulocoracoid, and the right radius and ulna. The bones are mostly dark brown and the principal matrix is dark red sandy mudstone with somewhat grayish mottling. The remains were collected in 2008 by two of us (DL and HY) along with the field team from the Fossil Research and Development Center, Gansu Provincial Bureau of Geo-exploration and Mineral Development. The localities that produced *Yongjinglong*, *Daxiatitan*, and *Huanghetitan liujiaxiaensis* are all near the town of Zhongpu (Fig. 1). The *Yongjinglong* locality, beside the G75 Highway, is less than a kilometer from the quarries that produced *Daxiatitan* and *Huanghetitan liujiaxiaensis*. This new specimen adds to the growing number of recent Chinese sauropod finds and highlights the increasing understanding of the diversity of Titanosauriformes in the Early Cretaceous of Asia.

## Materials and Methods

### Field Methods and Preparation

The holotype specimen described in this report was recovered by the field crew of the former Fossil Research and Development Center of the Third Geology and Mineral Resources Exploration Academy of Gansu Province for excavation and preparation of the specimens from the Early Cretaceous Hekou Group of the Lanzhou-Minhe Basin in southeastern Gansu Province, China in 2008. The team included Mr. Tao Wang and Mr. Huang and other workers, who carried out fieldwork and preparation. The specimen was recovered as a result of traditional, systematic field prospecting in sediments of the Hekou Group that had previously yielded a fauna comprising of sauropods and other dinosaurs. The fossils were jacketed in the field using standard plaster and burlap, and transported by truck to the Gansu Geological Museum in Lanzhou, where the fossils were extracted from their jackets and removed from the enclosing matrix using small pneumatic air scribes and dissecting needles. All specimens are curated in the collections of the Gansu Geological Museum, where they are available for comparative study to qualified researchers.

### Nomenclature Acts

The electronic edition of this article conforms to the requirements of the amended International Code of Zoological Nomenclature, and hence the new names contained herein are available under that Code from the electronic edition of this article. This published work and the nomenclatural acts it contains have been registered in ZooBank, the online registration system for the ICZN. The ZooBank LSIDs (Life Science Identifiers) can be resolved and the associated information viewed through any standard web browser by appending the LSID to the prefix “http://zoobank.org/”. The LSID for this publication is: urn:lsid:zoobank.org:pub:37C5C4B6-2009-462C-9FE4-9A9C3C9A3CBC. The electronic edition of this work was published in a journal with an ISSN, and has been archived and is available from the following digital repositories: PubMed Central, LOCKSS [http://www.lockss.org].

### Laminae and Fossae Terminology

The terminology of laminae and fossae in this study follows the nomenclature system of Wilson [20]–[21] and Wilson et al. [22]) with the exception that the terms “anterior, posterior” and their cognates are replaced by “cranial, caudal” and their cognates as required by adoption of standardized anatomical nomenclature for tetrapods, that is Nomina Anatomica Veterinaria (NAV) and the Nomina Anatomica Avium (NAA).

## Results

### Systematic Paleontology

Dinosauria Owen, 1842.
  Saurischia Seeley, 1887.
    Sauropoda Marsh, 1878.
      Macronaria Wilson & Sereno, 1998.
        Titanosauriformes Salgado, Coria, & Calvo, 1997.
          Somphospondyli Wilson & Sereno, 1998.
            Titanosauria Bonaparte & Coria, 1993.
*Yongjinglong datangi* gen. et sp. nov. urn:lsid:zoobank.org:act:D1D4479A-0BB7-454B-A62A-DDF5F808A09C.

### Etymology

The generic name “*Yongjing*,” from ancient Chinese, refers to Yongjing County, which is close to the fossil location of the new sauropod and which also yields numerous dinosaur track fossils; and “*long*,” meaning dragon, all in Chinese. The specific name “*datangi*” refers to the Tang dynasty and also honors Mr. Zhi-Lu Tang from the Institute of Vertebrate Paleontology and Paleoanthropology, Beijing for his numerous contributions to the research of dinosaurs.

### Holotype

GSGM ZH(08)-04 (Gansu Geological Museum, Gansu Province, China), comprising three teeth, eight presacral vertebrae including one caudal cervical vertebra, four cranial dorsal vertebrae, and three articulated middle dorsal vertebrae, one fragmentary dorsal rib and left scapulocoracoid as well as a right ulna and radius.

### Type Locality and Horizon

The location of *Yongjinglong* beside the G75 Highway is less than a kilometer from the quarries of *Daxiatitan* and *Huanghetitan liujiaxiaensis*, which are all located in the southeastern part of the Lanzhou-Minhe Basin (Fig. 1), Gansu Province, P. R. China; upper Hekou Group, Lower Cretaceous (Editorial Committee of Chinese Stratigraphic Standard: Cretaceous 2000).

### Diagnosis

Long-crowned, spoon-shaped premaxillary tooth; large, deep lateral pneumatic foramina (pleurocoels) spanning the entirety of the lateral surfaces of the cervical and cervicodorsal vertebrae; a complex “XI”- and “IX”-shaped configuration of laminae on left and right lateral surfaces of the articulated middle dorsal vertebrae, respectively; low unbifurcated neural spine that, along with the postzygapophyses, forms a cranially-pointing, triangular plate in at least one middle dorsal vertebra; very long scapular blade with exceptionally straight cranial and caudal edges.

## Description

### Teeth (Fig. 2; Table 1)

**Figure 2:**
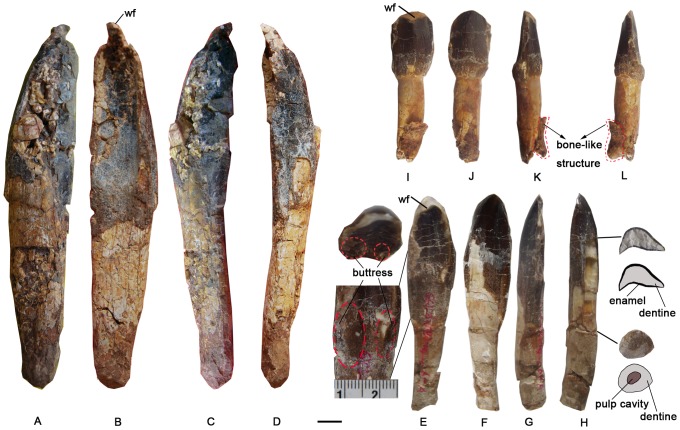
Teeth preserved in the holotype specimen of *Yongjinglong datangi* (GSGM ZH(08)-04). Labial, lingual and two lateral views of Tooth A (A, B, C, and D), of Tooth B (E, F, G and H), with crown and root cross-sectional views, a close-up of photo for the buttresses and interpretive outline drawings (see E and H), and of Tooth C (I, J, K, and L); red dashed lines in K and L delineate show a bone-like structure adhered to the tooth (K and L). Scale bar equals 10 mm. **Abbreviation: wf**, wear facet.

Three spatulate teeth are the only cranial elements preserved for the holotype. They are designated herein, from largest to smallest, Teeth A, B, and C. These three elements vary in size and shape presumably because of their positions in the jaw. Although all three are easily distinguished from each other, they are all spatulate (Fig. 2). All three have well-preserved crowns and roots.

Tooth A (Fig. 2A–D), the largest of the three teeth (Table 1), is presumed to be a mesial premaxillary tooth because of its size and the shape of the lingual-facing wear facet that is visible on the small, intact portion of its tip. The tooth has an elongate crown. The crown is asymmetrical in lingual view, with a more convex mesial side and a straighter distal side. The crown is strongly concave in lingual aspect. The long axis of the crown–root junction appears to be oblique to the long axis of the tooth, but this may be a result of missing enamel. The enamel is fairly thin and has a somewhat wrinkled surface texture that is oriented apicobasally. The lingual side of the tooth bears no apico-basal ridge, but is strongly concave. The labial surface is convex and bears one narrow and deep apico-basal groove on its mesial edge and a second, wide but shallow groove on the distal edge. The maximum mesio-distal width of the crown is more than twice that of the slender root.

**Table 1:** Measurements (in mm) of the three teeth preserved in the holotype of *Yongjinglong datangi* (GSGM ZH(08)-04).

Items	Tooth A	Tooth B	Tooth C
Total length	142	85	59.5
Maximum crown length	81	51	28
Maximum root length	61	34	31.5
Maximum mesiodistal crown width	20.6	19	17
Maximum mesiodistal root width	23	14	10
Maximum labiolingual crown width	19.8	13	11.5
Maximum labiolingual root width	12.7	12	11
Circumference at mid crown	85	48	32
Circumference at mid root	58	39.5	30
SI	3.93	2.68	1.65
Log SI	0.59	0.43	0.22
Average SI	2.75		
Log Average SI	0.44		

Note: SI = slenderness indices (maximum crown length divided by maximum mesiodistal width).

Tooth B (Fig. 2E–H; Table 1) likely comes from the mesial maxillary region based on its moderate medium width and apicobasally shorter crown, as is seen in the mesial maxillary teeth in other spatulate-toothed sauropods. It has the appearance of a more typical, spatulate sauropod tooth, and lacks the very tall crown of Tooth A, as reflected in its SI (slenderness index) value (Table 1). It is just over half the length of Tooth A. Its crown is convex labially and concave lingually, but less so than in Tooth A. The maximum mesio-distal width of the crown is more than twice that of the slender root. In lingual view, the tooth has a prominent, triangular wear facet on the apex of the crown and the wear facet is capped by a very sharp labial lip. The crown is more symmetrical in lingual and labial views than Tooth A; the symmetry makes it difficult to distinguish the mesial edge from the distal edge. There are no apico-basal grooves on the crown. The long axis of the crown-root junction is perpendicular to the long axis of the tooth. The enamel of this tooth is slightly smoother than that of Tooth A. Two asymmetric lingual crown buttresses are prominent (Fig. 2E). Breaks permit cross-sectional views of the crown and the root (Fig. 2H) and show that the enamel tapers in thickness from mesial to distal along both lingual and labial surfaces of the dentine. The maximum labio-lingual width of the dentine is over four times that of the enamel. On the cross-section of the crown, no pulp cavity is evident. The root is circular in cross section (Fig. 2H). Its pulp cavity, for passage of blood vessels, nerves and associated soft tissues [23], is round and nearly half the diameter of the whole root. The tip of the root is missing.

Tooth C, which is less than half the length of Tooth A, has a triangular crown and an almost circular root that is sharply set off from its crown (Fig. 2I–L; Table 1). The crown is relatively short and parallel-sided in labial view and has a rectangular cross section. The lingual aspect is more shallowly concave than on the preceding two teeth, but bears a strong apicobasal ridge close to the base of the crown. A wide, shallow groove occupies nearly half of the labial surface. The apex of the crown is broadly rounded, in contrast to the previous teeth, the result of the heavy wear corroborated by a wide mesio-distal wear facet. The short crown, which is heavily worn and diamond-shaped, the lingual wear facet, and the basal cingulum all tentatively suggest that it is more a distal dentary tooth. The labial side bears a broad apico-basal ridge. In the distal part of the root, some bone-like structure is attached to the root (Fig. 2K, L).

The three teeth vary in shape, size, and slenderness indices (Table 1). The depths of the concavities on their lingual sides gradually decrease from Tooth A to Tooth C. The crown of Tooth A is 1.6 and 2.9 times longer, respectively, than Tooth B and Tooth C. The SI value of Tooth A is 1.5 and 2.5 times larger, respectively, than the other two teeth. The degrees of wear in the three teeth may imply that Tooth A may have been more recently replaced, while teeth B and C are older teeth.

### Vertebral Column (Figs. 3–9; Table 2)

**Figure 8:**
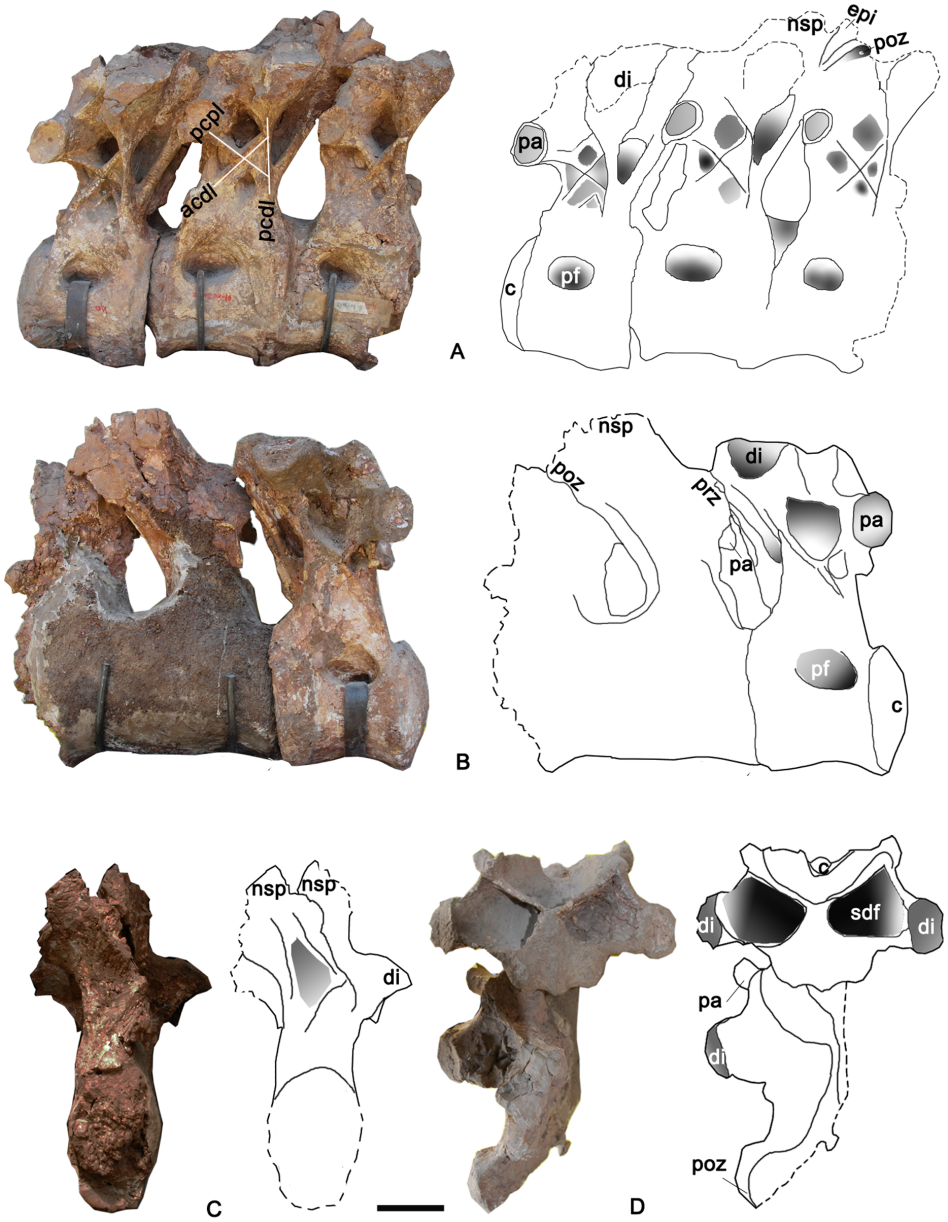
Three articulated caudal dorsal vertebrae of the holotype specimen of *Yongjinglong datangi* (GSGM ZH(08)-04). **A–D**, photographs and interpretive line drawings in **A**, left lateral; **B**, right lateral; **C**, caudal, and **D**, dorsal views. Scale bar equals 100 mm. Abbreviations as in Figs. 3–7.

**Figure 9:**
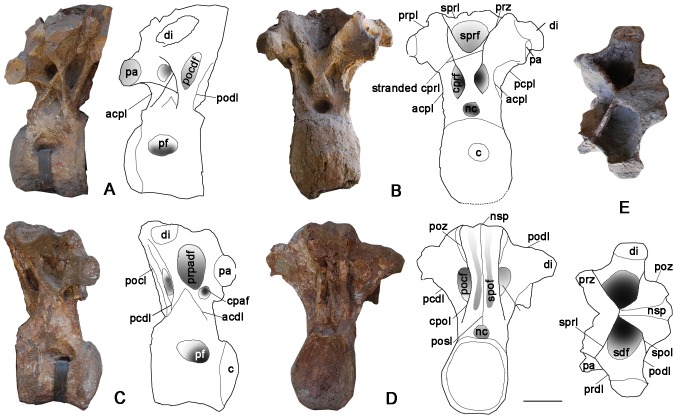
MD1 middle vertebra in the articulated dorsal series of the holotype specimen of *Yongjinglong datangi* (GSGM ZH(08)-04). **A–E**, photographs and interpretive line drawings in **A**, left lateral; **B**, cranial; **C**, right lateral; **D**, caudal, and **E**, dorsal views. Dotted lines indicate broken bone. Scale bar equals 100 mm. Abbreviations as in Figs. 3–8, plus: **acpl**, cranial centroparapophyseal lamina; **cpol**, centropostzygapophyseal lamina; **prpadf**, prezygapophyseal centroparapophyseal diapophyseal fossa; **prpl**, prezygaparapophyseal lamina; **spol**, spinopostzygapophyseal lamina; **pcpl**, caudal centroparapophyseal lamina; posf, postspinodiapophyseal fossa; **posl**, postspinal lamina.

The holotype of *Yongjinglong datangi* preserves eight opisthocoelous vertebrae: one caudal cervical, four cranial dorsal vertebrae, and an articulated sequence of three middle dorsal vertebrae. Seven of these vertebrae preserve relatively complete centra, but only the three articulated middle dorsals have relatively complete centra and neural arches with well-preserved features, as seen in lateral view (Figs. 8, 9A, C).

**Figure 3:**
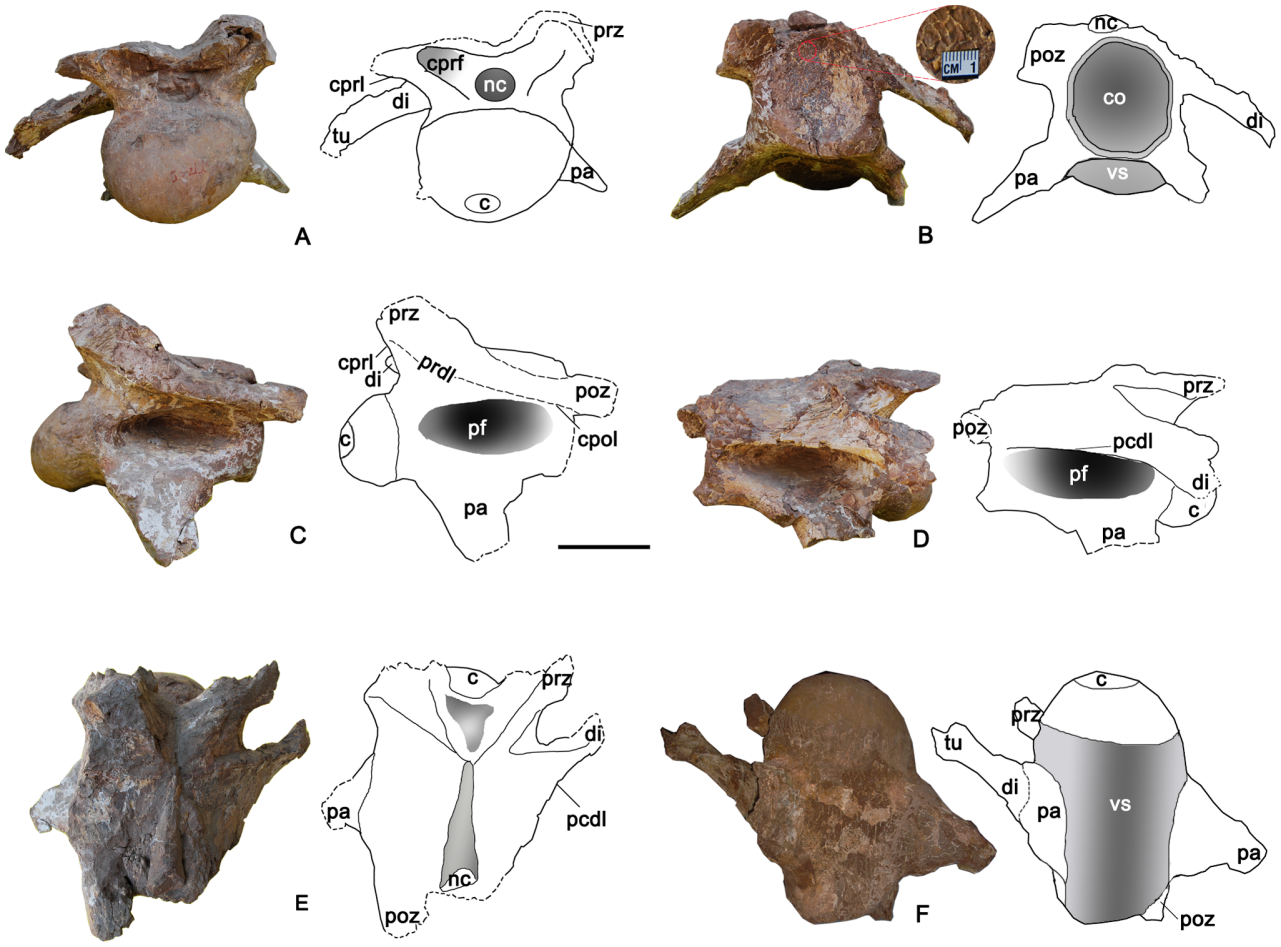
Caudal cervical vertebra of the holotype specimen of *Yongjinglong datangi* (GSGM ZH(08)-04). **A–F**, photographs and interpretive line drawings in **A**, cranial; **B**, caudal with a close-up photo to show the pneumaticity on the bottom of the cotyle; **C**, left lateral; **D**, right lateral; **E**, dorsal, and **F**, ventral views. Scale bar equals 100 mm. **Abbreviations**: **c**, centrum; **co**, cotyle; **cpol**, centropostzygapophseal lamina; **cprl**, centroprezygapophyseal lamina; **cprf**, centroprezygapophyseal fossa; **di**, diapophysis; **epi**, epipophysis; **nc**, neural canal; **pa**, parapophysis; **pcdl**, caudal centrodiapophyseal lamina(here “caudal” equals to “posterior” of Wilson [20]); **pf**, lateral pneumatic fossa or foramen; **poz**, postzygapophysis; **prdl**, prezygadiapophyseal lamina; **prz**, prezygapophysis; **tu**, tuberculum; **vs**, ventral surface.

### Caudal Cervical Vertebra (Fig. 3A–F; Table 2)

The caudal cervical vertebra has a well-preserved centrum and part of the neural arch, but lacks most of the neural spine and the postzygapophyses. Fragments of the tuberculum and capitulum are fused with the respective extensions of the diapophyses and parapophyses; this fusion is more obvious on the right side. The total combined height of the centrum and the preserved neural arch is not large (150 mm). The centrum is axially short, slender, and typically opisthocoelous. It bears a well-developed, hemispherical cranial condyle. The condyle is axially elongate and half the length of the centrum, from which it is strongly set off. The condyle is dorsoventrally compressed and asymmetrical in lateral view, with the ventral portion more bulbous than its dorsal counterpart. The caudal articular cotyle lacks the outer bone edge and appears as a shallow, concave surface with a distinctive camellate texture; the average diameter of a chamber is 5 mm and the thickness of the walls is less than 1 mm on average. The height and width of the cotyle are subequal. The ventral surface of the centrum is shallowly concave and lacks a keel. A deep, undivided lateral pneumatic foramen occupies the entire lateral surface of the centrum; its deepest (95 mm) point is close to cranial edge of the centrum. The lateral foramen may connect to its opposite counterpart. The upper margin of the foramen merges smoothly into the ventral margin of the neural arch. The ventral margin of the foramen is continuous with the base of the parapophysis; no fossa invades the dorsal surface of either parapophysis. The left parapophysis is better preserved than the right. The cranial base of the parapophysis is adjacent to the caudal edge of the condyle. The base of the parapophysis is remarkably long, spanning four-fifths of the ventrolateral margin of the centrum. The cranial edge of the parapophysis projects caudolaterally and the caudal edge projects craniolaterally; the two edges merge distally to form a tapered rib articulation.

The preserved part of the neural arch is relatively low, only half the height of the centrum. The neural spine is missing, so its height and orientation cannot be determined. The prezygapophyses are supported from below by single, stout centroprezygapophyseal laminae (cprl); no pre-epipophyses are present. The preserved parts of the prezygapophyses are low and oriented dorsolaterally in cranial view. The shapes of the articular facets of the prezygapophyses are unclear due to damage. The caudal part of the neural arch is damaged; only a fragment of the left postzygapophysis remains and it extends beyond the caudal margin of the centrum without evidence of an epipophysis. The diapophysis lies near the neurocentral junction and projects cranially and ventrolaterally over the lateral margin of centrum. The attached tuberculum of the rib, which appears as a lateral continuous part of the diapophysis, has been largely weathered away and exposes a cross section of the rib, which has a distinct, camellate texture internally. In lateral view, parts of the cprl, the posterior centrodiapophyseal lamina (pcdl), and the centropostzygapophyseal (cpol) laminae combine to form a long, continuous plate that tapers caudally. The neural canal is subcircular; its width and height are 55 mm and 45 mm, respectively. A slender, needle-shaped structure is visible in dorsal view that begins above the caudal cotyle and narrows cranially. It is composed of matrix and represents an infilling of the neural canal.

### Dorsal Vertebrae (Figs. 4–9; Table 2)

Seven dorsal vertebrae are preserved. Among them, four are identified as the cranial dorsal vertebrae on the basis of the positions of their parapophyses. The presumed first cranial dorsal is referred to as Dv1; the remaining three are referred to as Cranial DvA, DvB and DvC. The remaining three articulated vertebrae (MD1–3) are identified as middle dorsal vertebrae based on the sizes, shapes, and relative positions of their parapophyses and diapophyses.

### First Dorsal Vertebrae (Dv1) (Fig. 4A–F; Table 2)

**Figure 4:**
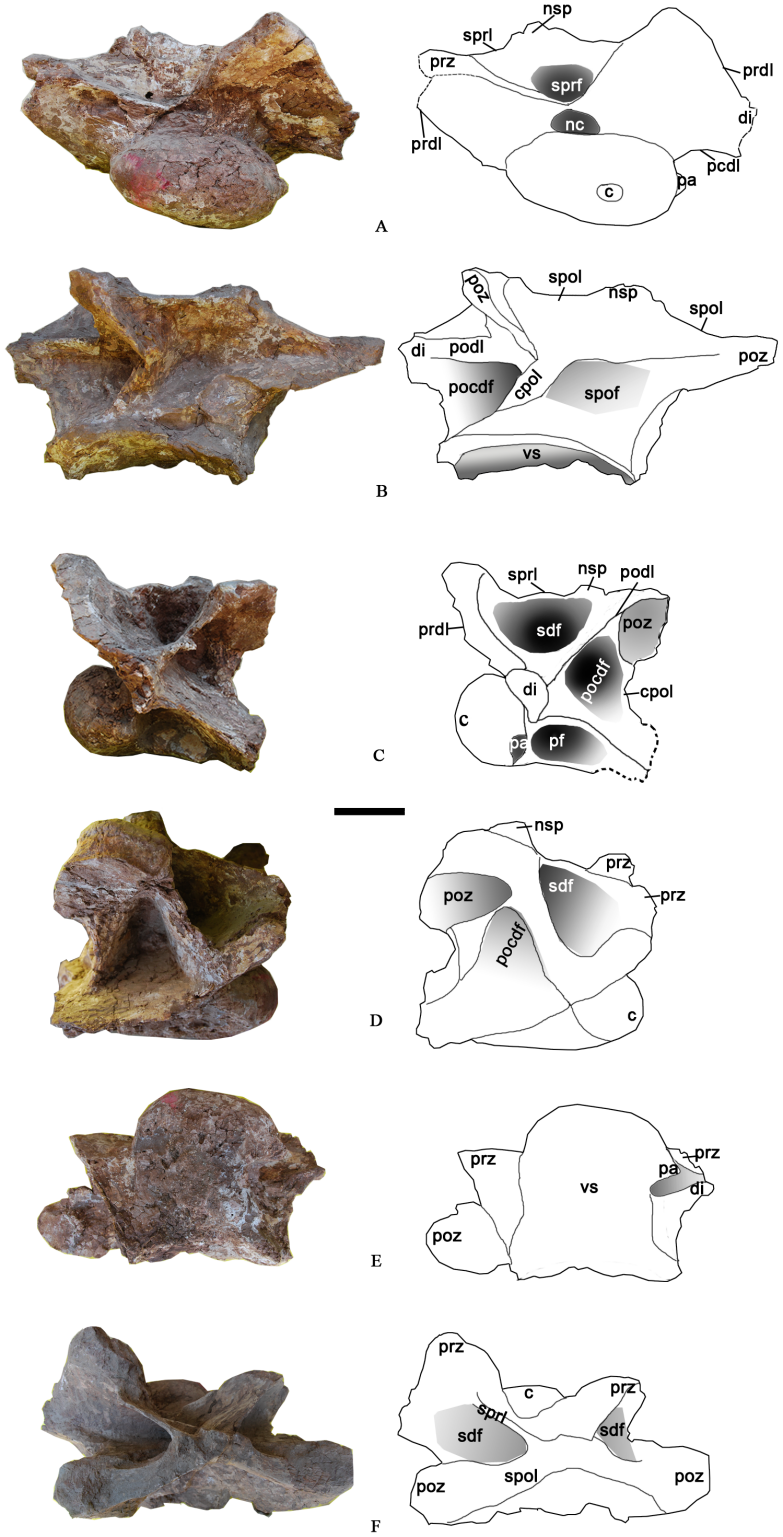
The relatively complete first dorsal vertebra of the holotype specimen of *Yongjinglong datangi* (GSGM ZH(08)-04). **A–E**, photographs and interpretive line drawings in **A**, cranial; **B**, caudal; **C**, left lateral; **D**, right lateral, and **E**, ventral views. Scale bar equals 100 mm. Abbreviations as in Figure 3, plus: **nsp**, neural spine; **pocdf**, postzygapophyseal centrodiapophyseal fossa; **podl**, postzygadiapophyseal lamina; **sdf**, spinodiapophyseal fossa; **sprl**, spinoprezygapophyseal lamina; **sprf**, spinoprezygapophyseal lamina fossa.

A massive vertebra preserves a parapophysis on the left lateral side of the centrum and has a relatively higher-positioned neural spine compared to the caudal cervical vertebra; it is here identified as the first dorsal vertebra. It comprises most of the centrum and neural arch. The ventral margin of the centrum is heavily damaged, but it retains the complete condyle. In cranial view, the condyle is dorsoventrally compressed and strikingly deformed as an asymmetric hemisphere: the left side is more expanded than the right side. Remarkably, the axial length of the bulbous condyle is half that of the rest of the centrum. As on the caudal cervical vertebra, the lateral pneumatic foramen occupies the entire lateral surface of the centrum and it shallows from cranial to caudal. Also as on the caudal cervical, the margin of the lateral pneumatic foramen coincides with the ventral surface of the pcdl. The ventral surface of the centrum appears to be nearly flat; it is damaged, but lacks any evidence of either a concavity or a ventral keel. In caudal view, the camellate texture on the cotyle surface comprises bony walls less than 1 mm in diameter (close-up in Fig. 4B).

The neural arch is slightly taller than its centrum, but is expanded laterally far more than in the caudal cervical vertebra. The neural spine is not well-preserved, but its base forms a small bump at the intersection of the spinoprezygapophyseal laminae (sprl) and the spinopostzygapophyseal laminae (spol) (Fig. 4A, B). The spol are robust and thick, but the sprl are virtually paper thin. The left prezygapophysis extends cranially beyond the condyle; its articular facet is rectangular and slightly convex transversely. The angle between the prezygapophyses is approximately 130° in cranial view (Fig. 4A). The left postzygapophysis bears a strong, ventrolaterally oriented articular facet, which is rectangular with a 135 mm-long major axis and 65 mm-long minor axis. In lateral view, the caudal margin of the postzygapophysis does not extend beyond the caudal edge of the centrum (Fig. 4C, D). The maximum distance between the dorsal tips of the postzygapophyses is nearly 460 mm, and the angle between them is close to 130°. The left diapophysis retains an airfoil-like cross section with a dorsoventral long axis. In lateral view, the prezygadiapophyseal lamina (prdl) is not thick but is wing-shaped. The postzygadiapophyseal laminae (podl) and the prezygadiapophyseal laminae (prdl) join to form a V-shaped structure in lateral view. The four laminae (prdl, sprl, podl, and spol) surround a large spinodiapophyseal fossa (sdf) that is 90 mm deep (Fig. 4B).

### Cranial Dorsal Vertebrae (Figs. 5–7; Table 2)

**Figure 5:**
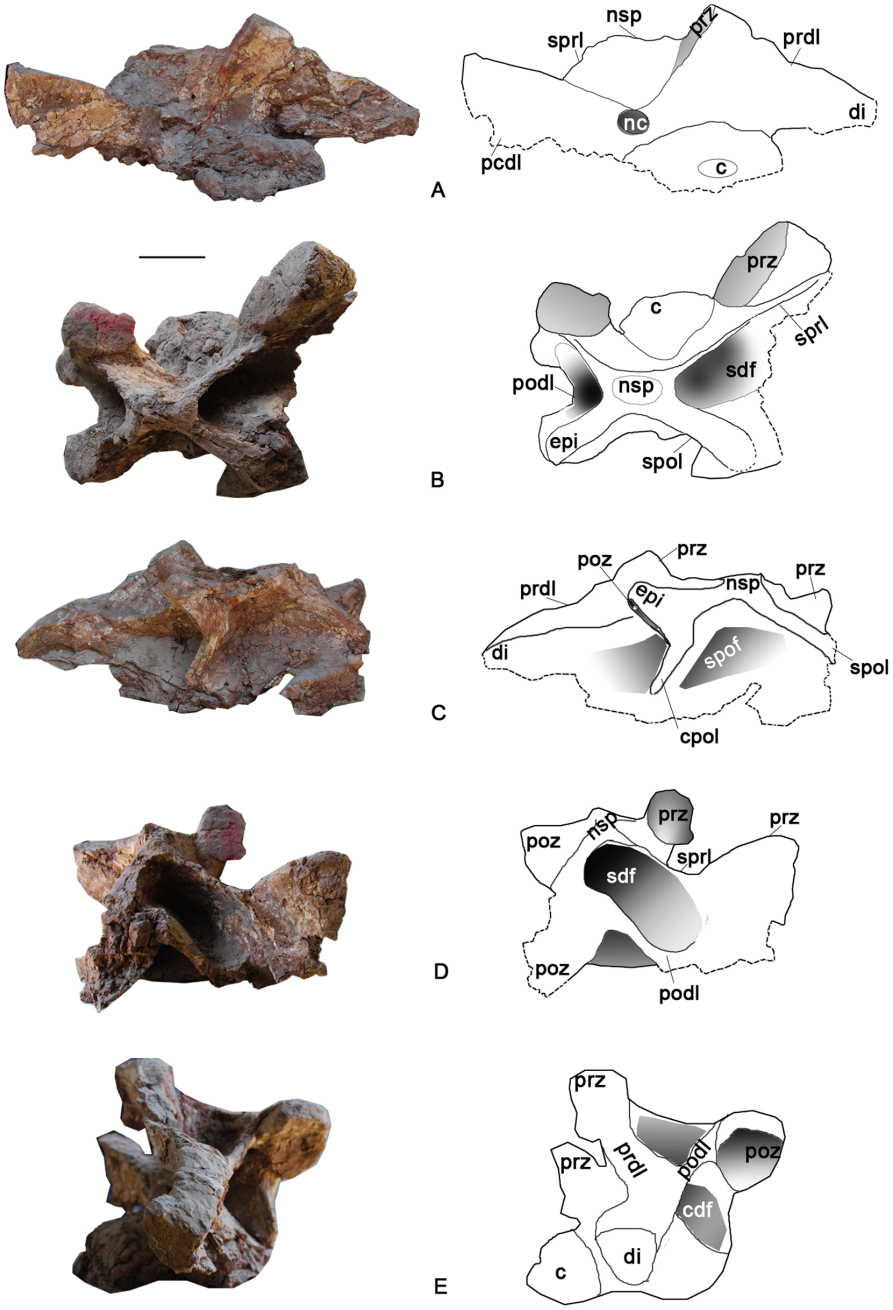
Cranial dorsal vertebra DvA of the holotype specimen of *Yongjinglong datangi* (GSGM ZH(08)-04). **A–E**, photographs and interpretive line drawings in **A**, cranial; **B**, dorsal; **C**, caudal; **D**, right lateral, and **E**, left lateral views. Scale bar equals 100 mm. Abbreviations as in Figs. 3 and 4, plus: **spof**, spinopostzygapophyseal fossa.

Cranial DvA is a heavily damaged and fragmentary vertebra (Fig. 5A–E; Table 2). Its centrum preserves only the dorsal portion of the condyle, but a partial neural arch shows several unusual features. This vertebra is identified as a cranial dorsal vertebra based on the similarity of its neural arch to that of Dv1. Lateral crushing has distorted the arch. The low, unbifurcated neural spine is wide transversely. The prezygapophyses are strikingly expanded laterally, with an angle between the articular facets of 120°. The articular facet of the left prezygapophysis is damaged; the right articular facet has a paddle-like surface with a 170 mm-long long axis and an 80 mm-long short axis. Both the spol and the sprl are slightly thinner than those of the caudal cervical vertebra. The right diapophysis is missing; the left diapophysis projects ventrolaterally. Compared to the previous vertebra, the left diapophysis is more robust. The prdl is slightly longer and strikingly more expanded laterally, and the sdf is much deeper.

**Table 2:** Measurements (in mm) of vertebrae preserved in the holotype of *Yongjinglong datangi* (GSGM ZH(08)-04).

Items	Centrumlength	Condyleheight	Condylewidth	Totalheight
Caudal Cv	210^+^	105	190	150
Dv1	200	–	–	–
Cranial DvA	160^+^	100^+^	–	130^+^
Cranial DvB	150	–	–	400
Cranial DvC	173	220	240	500
MD1	158	200	180	470^+^
MD2	178	190	–	530^+^
MD3	166^+^	190	–	500^+^

Notes: Cv = cervical vertebra; Dv = dorsal vertebra; “–” refers to missing data; “+” refers to estimated data. Centrum length here does not include the length of condyle ball; total height refers to the total length of neural arch and centrum.

**Figure 6:**
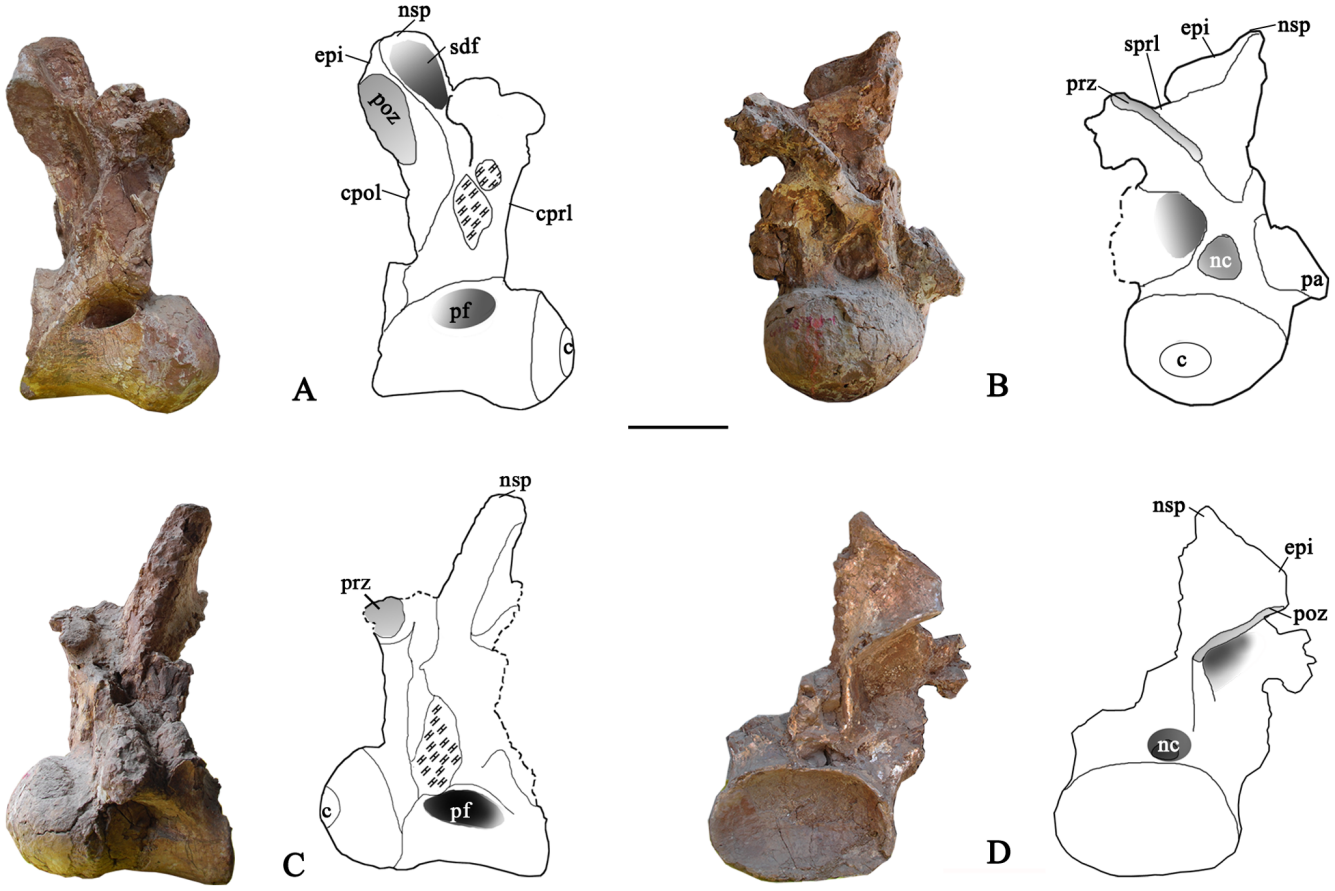
Cranial dorsal vertebra DvB of the holotype specimen of *Yongjinglong datangi* (GSGM ZH(08)-04). **A–D**, photographs and interpretive line drawings in **A**, right lateral; **B**, cranial; **C**, left lateral, and **D**, caudal views. Scale bar equals 100 mm. Abbreviations as in Figs. 3–5.

The other two dorsal vertebrae, labeled as Cranial DvB and Cranial DvC, are relatively well-preserved, though both lack parapophyses. The relatively higher neural arches indicate that they belong to the series of cranial dorsal vertebrae but caudal to Dv1 and Cranial DvA. But the arch of Cranial DvB (Fig. 6A–D; Table 2) is markedly lower than Cranial DvC and the three middle dorsal vertebrae. Hence we assign this Cranial DvB to a relatively cranial position of the trunk, but caudal to Cranial DvA. Cranial DvB bears a neural arch twice as high as its centrum. The centrum is short and slender and has a prominent condyle. The condyle is symmetrical in lateral view and closely similar to that of the caudal cervical vertebra. The height of the cotyle is slightly larger than that of the condyle. The ventral surface of the centrum is deeply craniocaudally concave and lacks a keel. The lateral pneumatic foramina are asymmetrical: the eye-shaped right one has an acute caudal margin and is situated on the dorsal margin of centrum, whereas the larger left one has a rounded caudal margin and is located in the middle of the centrum. Neither of the foramina is surrounded by a distinct fossa. The left prezygapophysis is completely missing; the right prezygapophysis is incomplete, but preserves most of the articular facet, which is less inclined than in the caudal cervical vertebra. The right postzygapophysis bears a large, elliptical articular surface faces caudoventrally and extends slightly beyond the caudal margin of the cotyle. A robust epipophysis on the dorsal side of postzygapophysis merges smoothly onto the neural spine. The partial neural spine is preserved primarily on the right side. The sdf is much larger and wider than that of the cervical vertebra, spanning all the way from its dorsal edge of the spol. The relatively large neural canal is teardrop-shaped with a rounded bottom.

**Figure 7:**
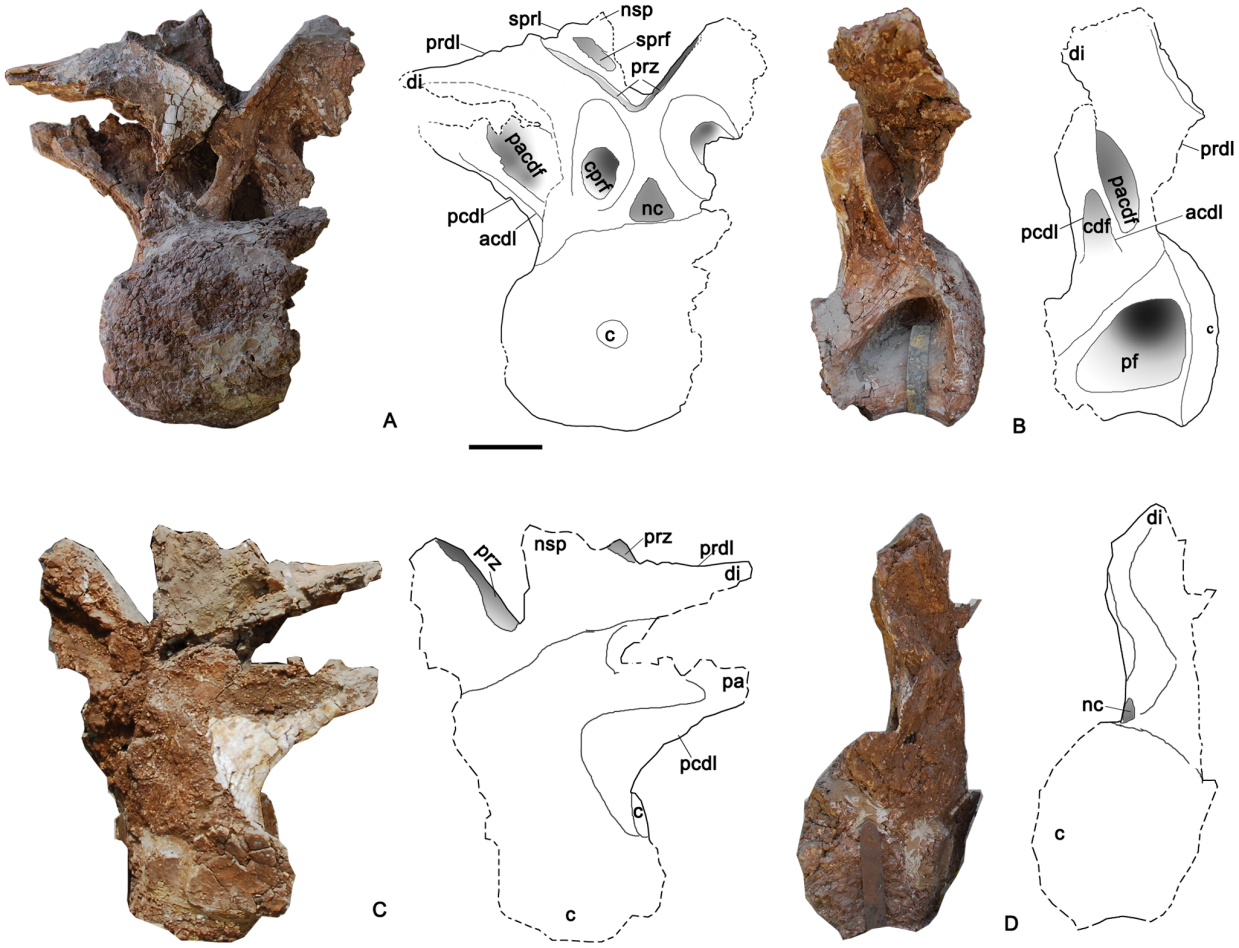
Cranial dorsal vertebra DvC of the holotype specimen of *Yongjinglong datangi* (GSGM ZH(08)-04). **A–D**, photographs and interpretive line drawings in **A**, cranial; **B**, right lateral; **C**, caudal, and **D**, left lateral views. Scale bar equals 100 mm. Abbreviations as in Figs. 3–6, plus: **acdl**, cranial centrodiapophyseal lamina (here “cranial” equals to “anterior” of Wilson [20]); **cdf**, centrodiapophyseal fossa.

The robust Cranial vertebra DvC (Fig. 7A–D; Table 2) is taller than Cranial DvA and DvB, but lacks parapophyses and other diagnostic characters that could reveal its exact position. It could be cranial dorsal vertebra 4 based on its high neural arch and the robust, compressed centrum. Cranial DvC retains part of the neural spine and centrum. Its large, deep lateral pneumatic fossa spans the lateral surface of centrum, reaching a depth of 160 mm close to the junction of the condyle and the centrum. In size and shape, the fossa resembles that of the cervical vertebra rather than those of the more caudal dorsals. The large fossa suggests a more cranial position, but the tall neural arch and massive centrum suggest a position farther along the dorsal vertebral column. The centrum also has a peculiar, large, and rather flat condyle in contrast to the previously described vertebrae. In lateral view, the condyle is axially compressed but very tall. The erect neural arch is markedly higher than the centrum and its preserved caudal edge does not overhang the plane of the centrum. Parts of the neural spine are present only on the right side. Both prezygapophyses are preserved; their articular facets have long axes oriented dorsolaterally. In cranial view, the neural canal is large and triangular, similar to those of the middle dorsal vertebrae (see below). The vertebra has a prominent centroprezygapophyseal fossa (cprf). The ventral parts of cranial and caudal centrodiapophyseal laminae (acdl and pcdl) are preserved but are damaged in their dorsal regions, where they should join the diapophysis. The preserved portion of the diapophysis is lateral to the prezygapophysis and hangs over the lateral surface of the centrum. In cranial view, the right cprf is deep and intact but the left one retains only the dorsal portion due to the missing part of the left cprl.

### Middle Dorsal Vertebrae (Figs. 8–9)

Three articulated dorsal vertebrae (from cranial to caudal: MD1, MD2, and MD3) are recognized as pertaining to the mid-dorsal column based on the sizes and shapes of the parapophyses and diapophyses, as well as the proximities of the processes to each other. Compared to the relatively low, transversely expanded neural arches of the cervical and cranial dorsal vertebrae, those of the middle dorsal vertebrae are much higher and closer to the midline. The lateral surfaces of these middle dorsals bear simple, undivided, and less-developed lateral pneumatic fossae, which are readily distinguished from the deep, prominent fossae in the cervical and cranial dorsal vertebrae. The three articulated middle dorsal vertebrae are rather massive. The three vertebrae are well-preserved on their left sides, but two of them lack neural spines. All are nearly equal to each other in the heights of the neural arches and the lengths of the centra (Figs. 8–9).

MD1 has a well-preserved neural arch and centrum (Fig. 9). The centrum is much lower than the neural arch and is subequal to it in length. In lateral view the compressed,camellate condyle is not very prominent and is less hemispherical than those of the cervical vertebra and Cranial DvA and DvB. However, the articular surface of the condyle is larger than that of the cervical vertebra and similar in size to those of the cranial dorsal vertebrae. In cranial view, the condyle is taller than it is wide and its greatest width is below the midpoint. The shape of the cotyle is congruent with that of the condyle. The lateral pneumatic foramen is surrounded by a shallow fossa that occupies nearly half of the lateral surface of the centrum, but the foramen is relatively shallower and smaller than those of the cervical vertebra and the cranial dorsal vertebrae. The ventral surface of the centrum is more strongly concave transversely than the cervical vertebra and the previous dorsal vertebrae.

The caudal part of the neural arch extends over the caudal rim of the cotyle. The neural spine is the only one well-preserved among all the preserved vertebrae of the holotype and is therefore of great importance for interpreting the relationships of this specimen. In dorsal view (Fig. 9E), the neural spine is fused with the postzygapophyses to form a cranially-pointing, trifid, triangular plate. This interesting trifid structure may reflect epaxial muscle tendon insertions associated with the postzygapophyses. The apex of the triangle is the summit of the neural spine, which is slightly higher than the diapophyses, craniolateral to the neural spine. The sprl, prdl, and spol surround a large, deep sdf. The sprl are slightly thicker than those of the cervical and cranial dorsal vertebrae; they subtend an angle of 110°. In cranial view, between the cprf of each side, lie clear, “stranded” [20] cprls dorsolateral to the neural canal (Fig. 9B). The prezygapophyses are slender and lie dorsal to the parapophyses. In cranial view, the parapophysis is situated high on the neural arch, lateral and slightly ventral to the prezygapophyses (Fig. 9B). In lateral view, the parapophyses project more cranially than the prezygapophyses (Figs. 9A, C). The parapophyses bear cupped, ovoid articular facets. In lateral view, the diapophysis lies farther caudodorsally than the parapophysis on the neural arch, below the neural spine; the articular facet on the right diapophysis is slightly concave and faces dorsolaterally. The right facet is larger than that of the parapophysis on the same side. A caudal centroparapophyseal lamina (pcpl) supports the diapophysis and connects with the acdl cranioventrally and pcdl craniodorsally. These three laminae thus frame a complex, “XI”-shaped configuration in left lateral view (or “IX”-shaped in right lateral view) (Fig. 8), in which two tiny, recessed accessory laminae lie below and parallel to the pcpl and acdl, respectively. The laminae of the XI-shaped complex surround four fossae of different sizes and shapes. In caudal view, a “stranded” postspinal lamina (posl) [20] is flanked by two parallel cpol without any sign of a hyposphenes; this absence is paralleled by the other two middle dorsal vertebrae. Ventrally, the posl bifurcates to form the roof of the neural canal. The neural canal is oval; its width and height are 50 mm and 40 mm, respectively.

The two following articulated dorsal vertebrae in the series, MD2 and MD3, are similar to MD1 in most respects (Fig. 8). The neural arches on both are noticeably taller than on MD1. The lateral pneumatic fossa of MD2 is larger than that of MD3. The XI-shaped complexes of laminae have more simplified fossae and sublaminae surrounded by the three main laminae mentioned above. The neural spines are too damaged to provide any further information. The postzygapophysis of MD2 lies at the level of the preserved neural spine of MD3, and its round, dorsal edge is attached tightly to the prezygapophysis of MD3. The left parapophysis of MD2 is slightly higher than those of the adjacent vertebrae, and it is slightly larger than that of MD3 on the same side.

### Rib (Fig. 10)

**Figure 10:**
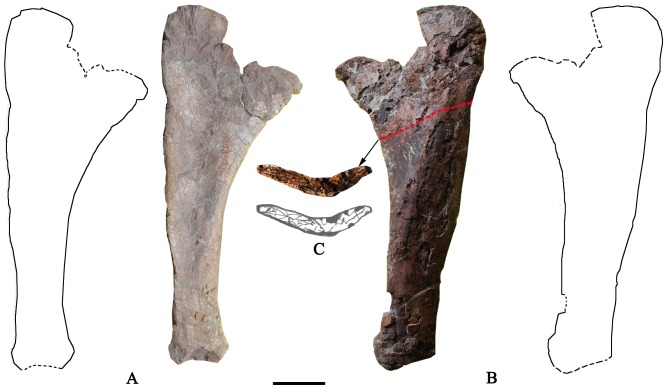
Proximal end of a dorsal rib of the holotype specimen of *Yongjinglong datangi* (GSGM ZH(08)-04). **A–C**, photographs and interpretive outline drawings in **A**, medial and **B**, lateral and **C**, the cross-section views. In views A and B the dashed outline drawings show the broken edges of the rib. In B, the red dashed line denotes the position of a natural break that exposes the interior structure of the element. In C, the grey area and line represent the bony wall or chamelle in the rib. Scale bar equals 100 mm.

The only costal element preserved in GSGM ZH(08)-04 is the proximal part of a heavy dorsal rib that is 678 mm long (Fig. 10). The maximum and minimum widths are 282 mm and 100 mm, respectively. The considerable distance between the capitulum and the fragmentary tuberculum suggests that the rib pertains to a cranial or middle dorsal vertebra. On the lateral surface, a robust ridge that begins near the caudoventral edge of the tuberculum runs diagonally across the midshaft and reaches the opposite edge distally (Fig. 10A). On both the ridge and the distal part of the rib, breakage has cleanly exposed a matrix infill of what could be either a simple medullary cavity or a pneumatic diverticulum. In medial view, a large groove occupies the whole middle surface of the preserved rib and gradually diminishes distally (Fig. 10B). The cross section of the plank-like rib through the robust ridge is triangular, with a breadth more than three time its thickness (Fig. 10C). In cross section the bone wall is thicker around the edge of the rib and thinner in the middle of the rib. The internal empty space, which is infilled with dark brown matrix (Fig. 10C), suggests the possibility of pneumaticity of ribs, though no pneumatic foramen is evident on the proximal end of the rib.

### Appendicular Skeleton

Preserved appendicular skeletal elements include the left scapulocoracoid, and the right ulna and radius.

### Scapulocoracoid (Fig. 11; Table 3)

**Figure 11:**
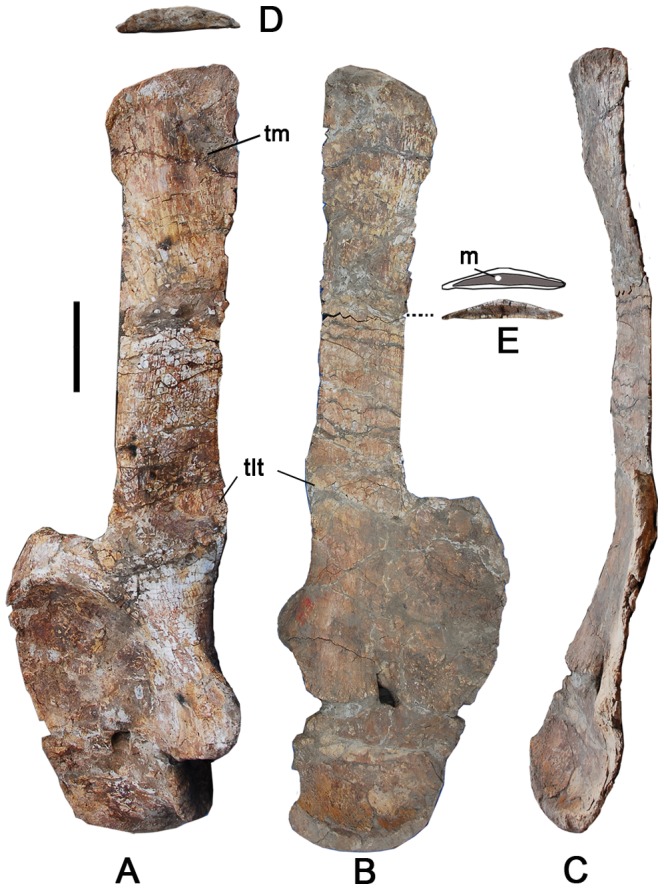
Left scapulocoracoid of the holotype specimen of *Yongjinglong datangi* (GSGM ZH(08)-04). **A–E**, photographs and interpretive line drawing in **A**, lateral; **B**, medial; **C**, cranial; **D**, distal and **E**, D-shaped cross-section of midshaft views. Scale bar equals 200 mm. Abbreviations: **tm**, ***M. teres major*** **; tlt**, *M. triceps longus* tubercle; **m**, medullary cavity.

The pectoral girdle is represented by a complete left scapulocoracoid of very distinctive form (we follow the convention of describing the scapulocoracoid as if it were oriented with its long axis vertical as shown in Figure 11; below we discuss evidence that the orientation was, *in vivo*, neither vertical nor horizontal ). The scapulocoracoid is very long, reaching 1940 mm in maximum length measured along the curvature (Table 3; Fig. 11). The scapula and coracoid are not fully co-ossified. The two bones are clearly fused cranially, while the caudal portion of the synostosis remains unfused, suggesting that the specimen could be a subadult. The synostosis is nearly perpendicular to the long axis of scapula. The surface around the caudal portion of the contact is very rugose, marking the origin site of the *M*. *coracobrachialis* [23]–[25]. The cranial margin of the scapular plate merges smoothly onto the coracoid except for a small piece missing at the synostosis. The scapula and coracoid contribute to the glenoid to form an obtuse angle (≈110°) with one another.

**Table 3:** Measurements (in mm) of pectoral girdle elements preserved in the holotype of *Yongjinglong datangi* (GSGM ZH(08)-04).

Items	Measurements items	Length
Scapulocoracoid	length (along curve)	1940
	length (straight)	1880
Scapula	length(along curve)	1580
	length(straight)	1550
	greatest breadth	680
	least breadth	215
	proximal breadth	290
	distal breadth	258
Coracoid	greatest length (dorsoventrally)	390
	greatest breadth (craniocaudally)	355
Glenoid	greatest expanse (long axis)	270

Note that directions of measurements are based on a vertical orientation of the scapula and coracoid.

### Scapula (Fig. 11; Table 3)

The scapula accounts for approximately 80% of the total length of scapulocoracoid (Table 3). It has a short proximal plate and a craniocaudally slender blade (Fig. 11A, B). In lateral view, the cranial edge of the acromion ridge of the plate extends straight dorsoventrally, without a marked dorsal angulation or hook. The thickness of the plate gradually increases from cranial to caudal and dorsal to ventral, starting at 20 mm along the craniodorsal edge and reaching a maximum of 190 mm close to the glenoid. The acromion bears a relatively prominent deltoid crest lying close to the dorsal margin of the plate; the angle between the longitudinal axis of the scapular blade and the oblique ridge of deltoid crest is about 58° degrees. This oblique ridge forms the dorsal border of a single, large fossa for the origin of *M*. *deltoideus scapularis* [24]. The scapular portion of the glenoid faces caudoventrally, has a deep, eye-shaped, concave surface, and exhibits slight medial beveling. Strikingly, the scapular blade is more than twice as long as the plate but only half as wide. The length of the blade is about 4.5 times its minimum width. The distances from the dorsal edge of the acromion to the ventralmost point of the glenoid and to the mid-point of the blade are subequal. The cranial and caudal margins of the blade are straight and parallel to each other (Fig. 11A, B). Through the middle of the blade, the cross section is roughly D-shaped, slightly concave medially and convex laterally. In the broken cross section, the outer rim of the blade is made of dense cortical bone, but the interior is spacious and filled with sediment, indicating a large medullary cavity (Fig. 11). Close to the proximal end of the scapular blade, on the lateral surface, a shallow fossa bordered by a ridge may mark the origin for the *M*. *scapulohumeralis* and *M*. *dorsalis scapulae* [23], [24]. Dorsal to the glenoid and the acromion, on the caudal edge of the blade, lies a low but prominent triangular process (Fig. 11A, B). This process is considered an origin site of *M*. *triceps longus* [24] and we here name it the *triceps longus* tubercle (tlt). The middle of the blade is the thinnest portion mediolaterally, in contrast to both the proximal and distal portions. The minimum breadth of the slender blade (215 mm) is one-seventh of the scapular length. No ridges are developed on the medial surface of the blade, but a single, prominent ridge appears on the lateral surface of the blade. The ridge lies along, and is oriented parallel to, the midline of the blade and spans nearly its whole length. The distal end of the scapular blade is slightly expanded caudally, but retains a D-shaped cross section (Fig. 11D). On the distal end of the lateral surface, another fossa marks the probable origin of the *M*. *teres major* [23], [24].

### Coracoid (Fig. 12; Table 3)

**Figure 12:**
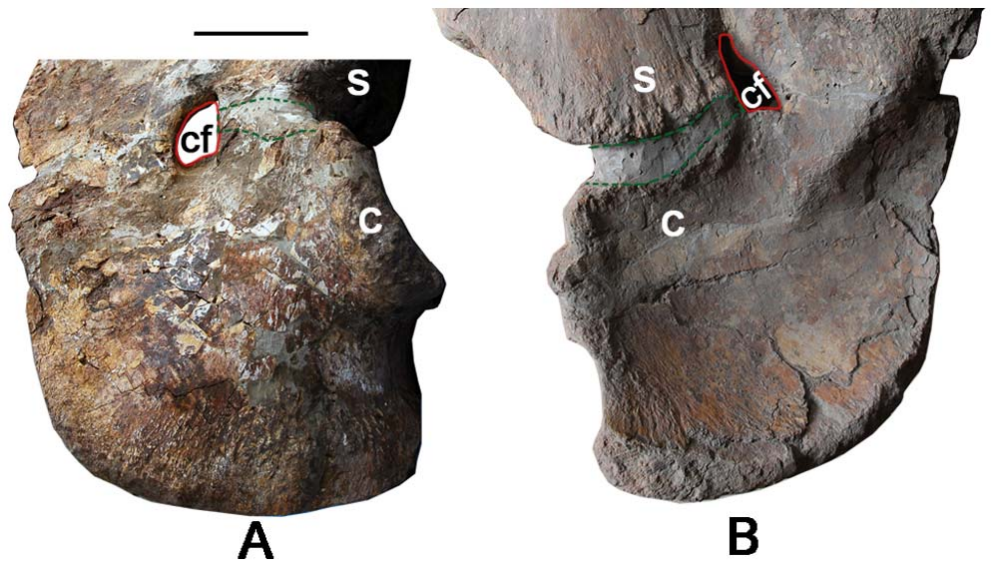
Coracoid of the holotype specimen of *Yongjinglong datangi* (GSGM ZH(08)-04). Elements in lateral (A) and medial (B) views. The red line denotes the coracoid foramen, and the green dashed lines demarcate the unfused region infilled with the grey matrix, Scale bar equals 100 mm. Abbreviations: **s**, scapula; **c**, coracoid; **cf**, coracoid foramen.

Compared to the extremely long scapula, the coracoid is a small, square plate, only one-fourth the length of the scapula (Figs. 11, 12). The dorsoventral length of the coracoid is subequal to its width but only half that of the scapular plate. The planar coracoid portion of the glenoid faces caudodorsally and is smaller in outline than the concave elliptical scapular half of the glenoid. The coracoid is partially fused with the scapula: the fusion of the two elements apparently proceeded from cranial to caudal and was arrested in mid-fusion. The unfused part (40 mm width dorsoventrally) is close to the glenoid cavity and is filled with grey matrix (Fig. 12A, B), suggesting incomplete ossification consistent with subadult status. On the lateral side, the large, nearly circular coracoid foramen, the passage for *N. supracoracoideus* [26] and the supracoracoid artery [27], is shared almost equally by the scapula and coracoid (Fig.12A). On the medial side, however, though the synostosis is somewhat difficult to discern, but it appears that most of the coracoid foramen penetrates the adjacent plate of the scapula (Fig. 12B); its broad base rests in the coracoid but the keyhole-shaped foramen extends dorsally onto the plate of the scapula.

### Right Ulna and Radius (Figs. 13–15)

**Figure 13:**
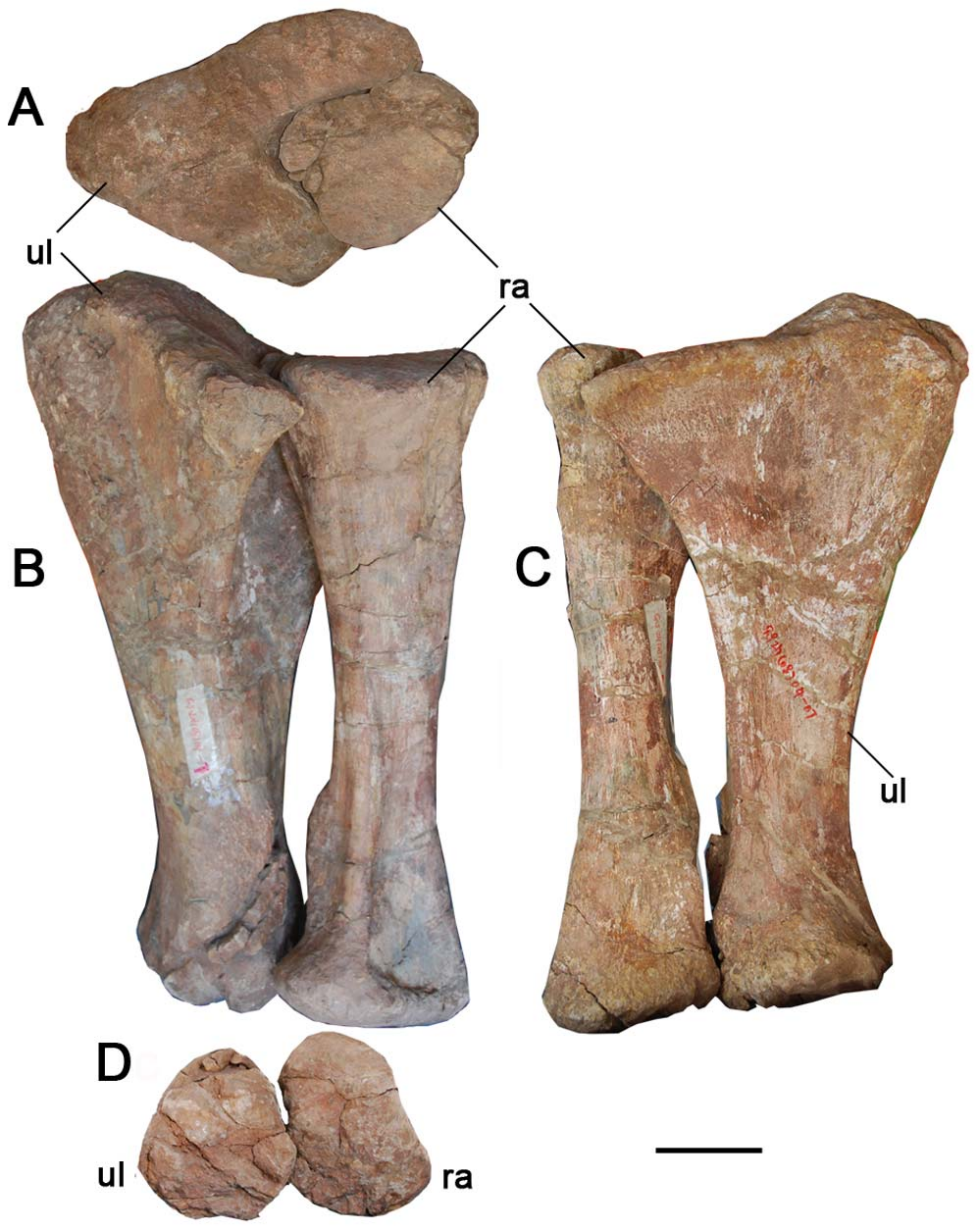
Right ulna and radius of the holotype specimen of *Yongjinglong datangi* (GSGM ZH(08)-04). Elements in proximal (A), medial (B), lateral (C), and distal (D) views. Scale bar equals 100 mm. Abbreviations: **ul**, ulna; **ra**, radius.

The right ulna and radius are well-preserved. The proximal end of the radius is mostly enclosed by the ulna. In proximal, lateral, and distal views, the two elements articulate tightly (Fig. 13).

### Ulna (Figs. 13–15; Table 4)

**Figure 14:**
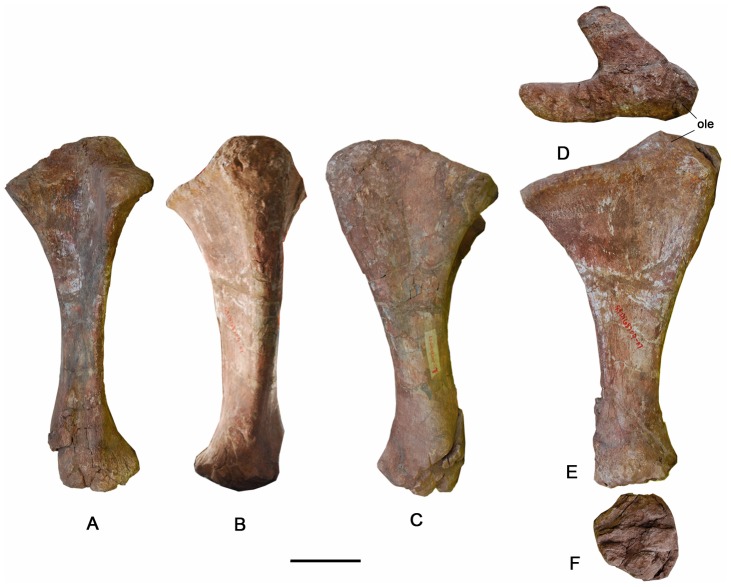
Right ulna of the holotype specimen of *Yongjinglong datangi* (GSGM ZH(08)-04). Elements in cranial (A), caudal (B), lateral (C), proximal (D), medial (E), and distal (F) views. Scale bar equals 100 mm. Abbreviation: **ole**, olecranon.

The right ulna is a robust element, though the proximal end is much more robust than the distal end (the proximal mediolateral width divided by the total length is 0.57). The proximal end is triradiate, with craniomedial, craniolateral, and caudal processes (Fig. 14), which form a well-developed notch or groove on the cranial margin for receiving the radius. In proximal view, the strongly concave craniomedial process is longer and more slender than the craniolateral process. The craniolateral process, however, is more elevated and slopes gently cranially. The craniomedial and craniolateral processes subtend an angle of 70°. The caudal process, which is capped proximally by the olecranon area that marks the site of insertion of the triceps muscles, is round and slightly higher than the craniolateral process. It tapers gradually to merge into the craniolateral process, but forms a sharp step at the junction with the craniomedial process. The step corresponds to the boundary between the cranial processes that articulate with the humerus and the non-articular olecranon. In cranial view, the lateral process is much higher than the medial one. A robust ridge extends distally along the proximocaudal margin of the ulna. Both the lateral and the medial surfaces of the proximal portion of the ulna are strongly concave (Fig. 14A). The triangular shaft of the ulna tapers to a rugose, ovoid, caudally expanded surface at the distal end, though the distal end that expands caudally. The lower half of the ulna presents two well-defined interosseous ridges that bound a strong radial groove into which the caudodistal end of the radius articulates (Fig. 14B). As with the radius, in cranial view the distal end of the ulna bears rugose texturing that may correspond to attachments of the *M*. *pronator quadratus* [24] or ligamentous tissues, such as a syndesmosis binding the ulna to the radius. The minimum circumference of the ulna lies slightly below the midshaft.

### Radius (Figs. 13–15; Table 4)

**Figure 15:**
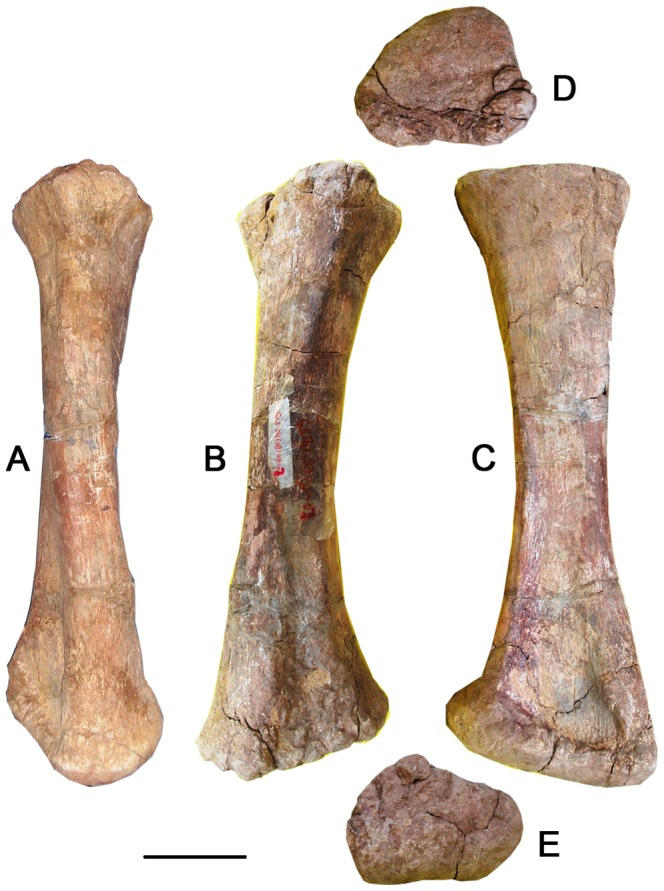
Right radius of the holotype specimen of *Yongjinglong datangi* (GSGM ZH(08)-04). Elements in cranial (A), caudal (B), lateral (C), proximal (D), and distal (E) views. Scale bar equals 100 mm.

The complete right radius is robust (proximal width divided by the total length is 0.30) and has a relatively straight shaft with expanded proximal and distal ends (Fig. 15). The proximal end is about as wide as the distal end (Figs. 14D, 15C). The triangular proximal end is slightly concave and the caudal border is contoured to fit into the cranial fossa or radial notch of the ulna (Figs. 13A, 15D). The cross section of the shaft is elliptical and slightly craniocaudally compressed. The minimum circumference lies at midshaft, and it is less robust than the ulna. The distal articular surface of the radius is oblique to the long axis of the shaft at an angle of 20° to the horizontal.

Three prominent ridges along the margins of the triangular bone define corresponding grooves or fossae on the distal shaft of the radius. The caudal side of the distal radius bears a pair of parallel ridges surrounding a sulcus that faces the cranial side of the ulna. The groove is for the insertion of the *M. pronator quadratus* or ligamentous tissues, such as a radio-ulnar syndesmosis. The more caudolateral and shorter of the two ridges is sharply set off from the radial shaft, while the longer and more caudomedial ridge tapers gradually to the distal end of the radius. The lateral ridge is the most robust of the three (Fig. 15B). On the lateral surface of the distal shaft of the radius it bears a tall, deep groove, which is about 15mm deep.

## Comparison of *Yongjinglong datangi* with Other Sauropods

### Teeth

The spoon-shaped premaxillary Tooth A has a long crown that is among the tallest of all sauropods thus far reported [28]–[33]. Asymmetric prominent lingual crown buttresses, such as on Tooth B, are also seen in *Euhelopus* [34] and an unnamed sauropod from eastern China [35]. The dental SI values may differ within the same individual for any sauropod. For instance, an individual of *Mamenchisaurus youngi* has higher SI values (maximum 2.9) in its premaxillary and mesial maxillary teeth and lower SI values (minimum 1.0) in its distal maxillary and dentary teeth [32]. The SI values of *Yongjinglong datangi* range from 3.93 to 1.65 and average 2.75 (Table 1) which is relatively higher than those of Euhelopodidae1, Euhelopodidae2 and Euhelopodidae4 (1.71, 1.90 and 2.15), but lower than that of Euhelopodidae3 described by Chure et al. (suppl1.pdf, [33]).

### Vertebrae

All of the preserved vertebrae, including condyles, are short (less than 300 mm long, individually). Their sizes are far smaller than those of the hyper-long-necked sauropods, such as *Daxiatitan binglingi*, *Sauroposeidon proteles* [36], [37], *Barosaurus lentus* [38], and *Supersaurus vivianae* [39], [40]. The juvenile specimen of *Yongjinglong* is estimated to be a medium-sized sauropod.

Cervical pneumatic fossae on the centra are common in sauropods. In western North American sauropods, such as *Suuwassea* [41], *Brachiosaurus* sp. [42](BYU 12866; BYU, Brigham Young University, Provo, UT, U.S.A), and *Barosaurus,* and also in *Galvesaurus herreroi* from Spain [43], they are sometimes present as several foramina separated by septa positioned within larger fossae. However, in known Asian sauropods, they are less developed and present as large, shallow, single fossae, especially in *Mamenchisaurus*, *Daxiatitan* and *Euhelopus*. *Yongjinglong* bears large, deep, simple foramina within fossae and they uniquely span nearly the entire lateral surfaces of the centra of its caudal cervical and cranial dorsal vertebrae, strikingly different in size and span from the aforementioned Asian sauropods. Eventually, the lateral pneumatic foramina become shallow in the middle dorsal vertebrae.

The cervical parapophyses in sauropods are usually short, small processes, as in *Camarasaurus* [32], [44] and *Diplodocus* [45]; they are less than one-third the functional length of the centra (i.e., excluding the condyle) in *Daxiatitan*, *Euhelopus*, *Phuwiangosaurus* [46], and *Apatosaurus* [47]. In *Malawisaurus* [48], *Alamosaurus* [49], and members of “Saltasaurini” (including *Saltasaurus* [50]), the parapophyses are close to 50% of the functional length of the cervical centra [19]. However, the parapophysis of *Yongjinglong* is about 80% of the length of the preserved caudal cervical centrum, longer than in all other sauropods and constituting an autapomorphy of *Yongjinglong*. The parapophysis is also dorsoventrally elongated, as in *Daxiatitan* and *Euhelopus*. The prominent, transversely expanded structure formed by the junction of the prdl and podl in the first dorsal vertebra surrounds a large, shallow fossa on the dorsal surface of the neural arch, similar to that seen in *Hudiesaurus* [51].

The centra of the articulated middle dorsal vertebrae of *Yongjinglong* are much smaller than those of *Daxiatitan* (nearly one-fourth the length of the longest one of *Daxiatitan*; L. Li, in preparation), but slightly stouter than those of *Euhelopus* (Table 2) ([1], table 2) and *Qiaowanlong*.

In *Yongjinglong*, the size, shape, and location of the parapophyses vary remarkably from the cervical series to the middle dorsals. From cervical to dorsal, the parapophyses transform from low, long plate-like structures on the ventral margin of the cervical centrum to high, ovoid processes located cranioventral to the diapophyses. The parapophyses of the middle dorsals are similar to those of the dorsals 6–10 in *Euhelopus* in both shape and position.

**Figure 17:**
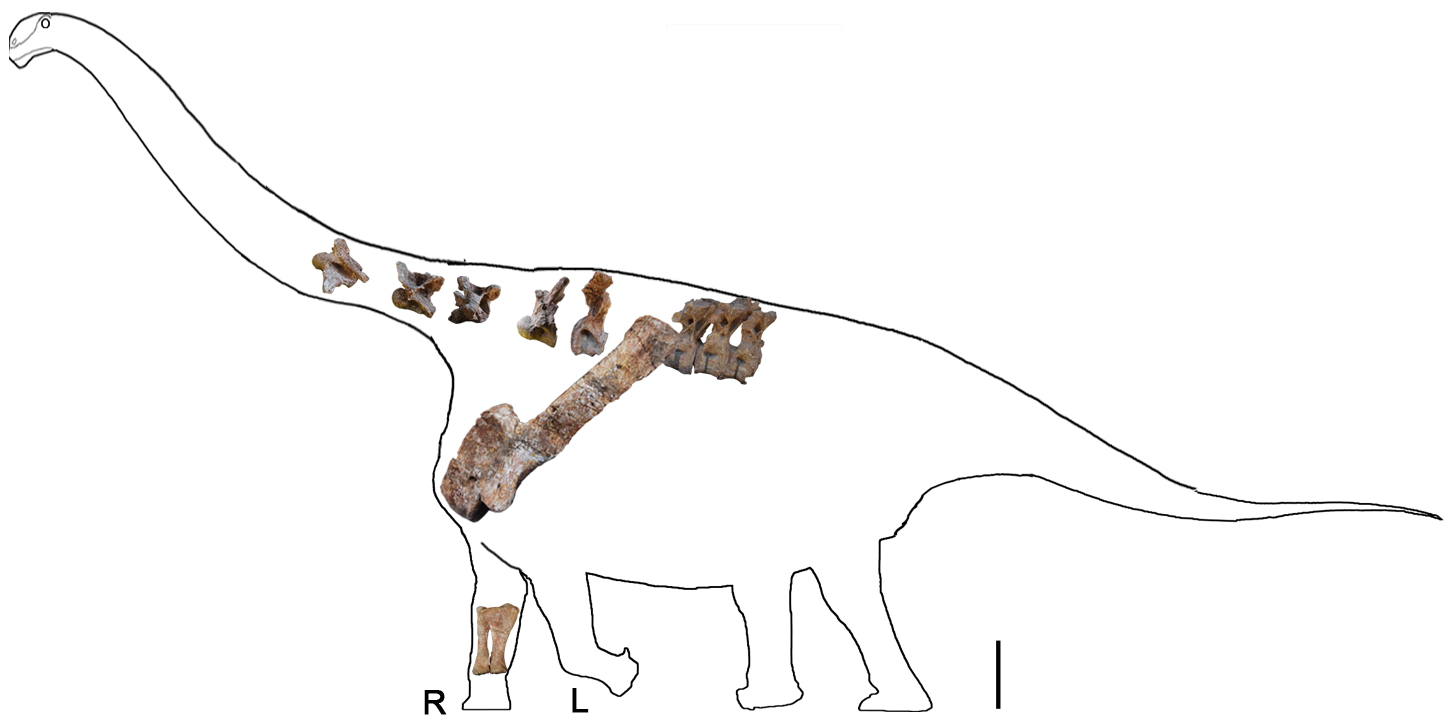
Skeletal reconstruction of the preserved postcranial elements of the holotype specimen of *Yongjinglong datangi* (GSGM ZH(08)-04). All elements are shown in left lateral view except the right ulna and radius which are in right medial view. Abbreviations: **R**, right; **L**, left. Scale bar equals 600 mm.

**Figure 18:**
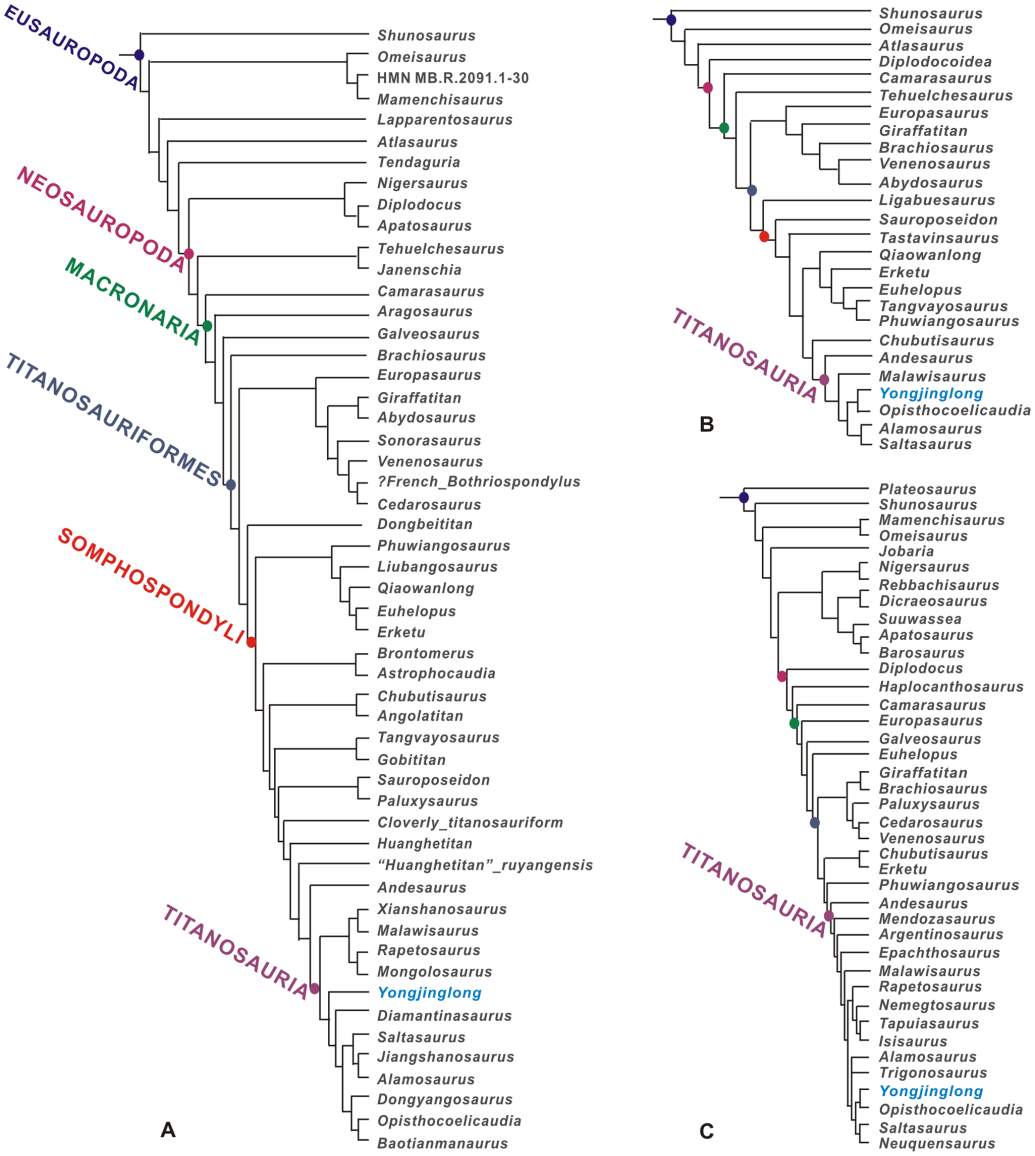
Strict consensus trees of *Yongjinglong datangi*. Phylogenetic hypothyses were obtained by TNT. A, Tree based on the data matrix of Mannion et[18] *Yongjinglong datangi* is basal to *Malawisaurus* and *Opisthocoelicaudia* but more derived than *Andesaurus*; B, Tree based on the data matrix of D’Emic [19]. *Yongjinglong datangi* is within Titanosauria and consititutes the sister taxon of *Opisthocoelicaudia*. C, Tree based on the data matrix of Carballido & Sander [61]. *Yongjinglong datangi* is recovered as sister taxa with *Opisthocoelicaudia*.

The lateral pneumatic fossae of the neural arches appear to be slightly larger in the dorsal vertebrae of *Yongjinglong* than the corresponding ones in *Euhelopus*. The prominent, autapomorphic, “XI”-shaped configuration of the diapophyseal and parapophyseal laminae on the left lateral aspects of the neural arches in the middle dorsals of *Yongjinglong* is similar to, but more elaborate than, those observed on the dorsal vertebrae of *Euhelopus* ([34], figs. 17, 18) with the extra, small laminae and fossae inside the XI structure. This complex is not developed in *Daxiatitan* and other described Asian sauropods. Because of incomplete preservation, the configuration of the neural spines cannot be determined in *Yongjinglong*; nevertheless, based on what is preserved, there is no evidence of bifurcation of the neural spines as seen in *Mamenchisaurus*, flagellicaudatans and putative Euhelopodidae. The only well-preserved neural spine in *Yongjinglong* (MD1) is fused with the epipophyses and postzygapophyses to form a transverse “trifid” structure in dorsal view. This trifid structure is not the same as in *Euhelopus* in which the trifid neural spine is composed of paired metapophyses and a median tubercle ([34], fig. 15). Furthermore, in *Euhelopus* the apex of the trifid spine projects caudally, the opposite of the orientation in *Yongjinglong*.

### Scapulocoracoid (Fig. 16)

**Figure 16:**
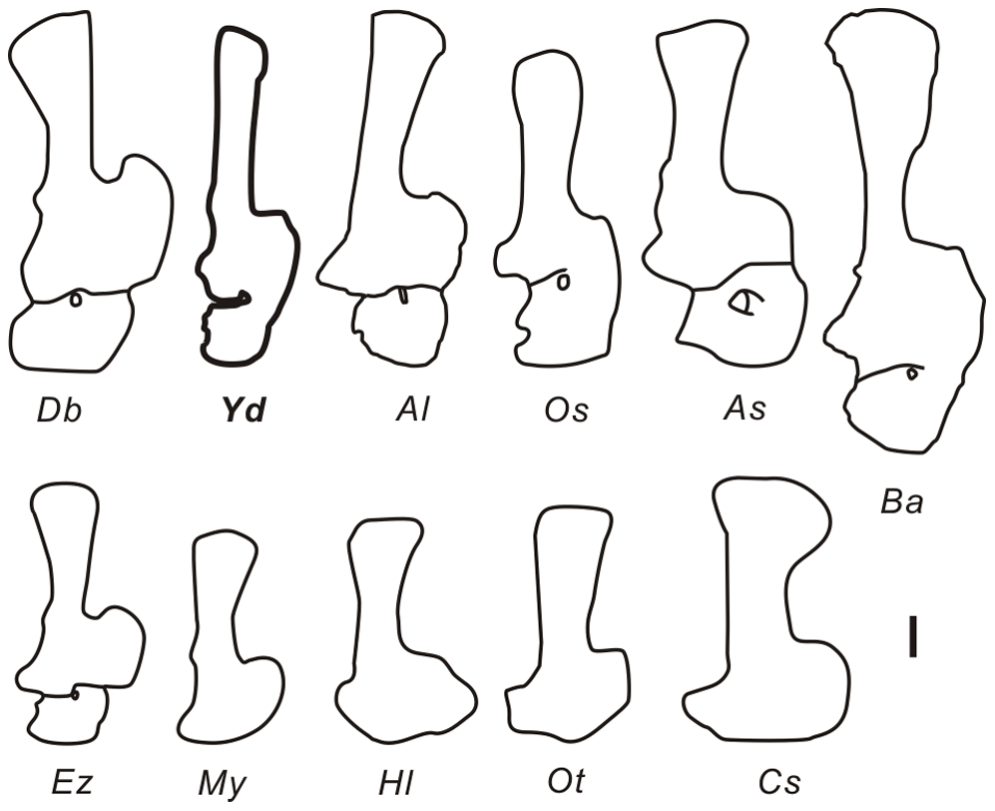
Comparison of the morphologies of 11 select scapulocoracoids. *Db–Daxiatitan binglingi* (modified from [14]), *Ez–Euhelopus zdanskyi* (modified from[2]), *Xd–Yongjinglong datangi*, *Al–Apatosaurus louisae* (modified from [47]), *As–Alamosaurus sanjuanensis* (modified from [49]), *Ba–Brachiosaurus altithorax* (modified from [52]), *Os–Opisthocoelicaudia skarzynskii* [53], *My–Mamenchisaurus youngi* (modified from [32]), *Hl–Huanghetitan liujiaxiaensis* (modified from [13]), *Ot–Omeisaurus tianfuensis* (modified from [4]) and *Cs–Camarasaurus supremus* (modified from [28]). Scale bar equals 200 mm.

Despite the comparatively small size of the individual, the left scapulocoracoid is much longer in *Yongjinglong* than in most sauropods except *Daxiatitan*, *Alamosaurus*, *Camarasaurus*, *Apatosaurus, Supersaurus*, and *Brachiosaurus* sp. [52] (Fig. 16; Table 5). *Yongjinglong* has a higher SCI value (ratio of the maximum length of scapula divided by that of the coracoid) than any sauropods except for *Supersaurus*, *Apatosaurus*, *Barosaurus* and *Omeisaurus* (Table 5). Its relatively long, narrow scapular blade, which accounts for more than one-half of the entire length of the scapulocoracoid, is slightly longer than those of the much larger individuals of *Supersaurus* and *Apatosaurus*. It is proportionally one of the longest blades among sauropods. The caudal edge of the blade, similar to that of *Apatosaurus*, is remarkably straight, rather than concave as in other sauropods (Fig. 16), and the cranial side is nearly parallel to it except for a very slight expansion near the distal end. This slight distal expansion in *Yongjinglong* contrasts with the marked expansions of the distal ends of the scapulae in most sauropods (Fig. 16). Typical sauropod scapulae have a single, subtriangular tlt, though the Asian sauropod *Opisthocoelicaudia* [53] lacks one; a second tubercle appears in *Alamosaurus* and *Sauroposeidon proteles* (UM20800) [54]. The tubercles reflect the attachment of *M. triceps longus*. The presence of the feature was first used as a character in the phylogenetic analysis of *Chubutisaurus insignis* ([55], in which it was called the “ventromedial process”); it was then coded as “the subtriangular process at the cranioventral corner of the scapular blade” by Mannion et al. [18]. The subtriangular tlt of *Yongjinglong* is not as prominent as that of *Daxiatitan*, but closer to those of *Euhelopus* (Fig. 16), *Ligabuesaurus* [56] and *Chubutisaurus*. In *Yongjinglong*, the scapular plate is not as expanded as in *Daxiatitan*, *Huanghetitan liujiaxiaensis*, or *Opisthocoelicaudia.* The size of the plate is closer to that of *Euhelopus*, but the shape is different: the glenoid region is prominent in *Euhelopus* but relatively weak in *Yongjinglong*.

**Table 5:** Comparison of scapulocoracoid lengths (in mm) among select sauropods.

Taxon	Specimen No.	L(SC)	L(S)	L(C)	SCI	Resource
*Supersaurus vivianae*	BYU 9025	2350	1920	430	4.47	[40]
*Apatosaurus louisae*	CM 3018	2020	1640	370	4.43	[47]
*Barosaurus lentus*	AMNH 6341,	1597	1300	297	4.38	[38]
*Omeisaurus tianfuensis*	T5701	1700	1380	320	4.31	[4]
*Yongjinglong datangi*	GSGM ZH(08)-04	1940	1580	390	4.05	This study
*Brachiosaurus* sp.	BYU 9462	2500	1990	510	3.90	[52]
*Mamenchisaurus youngi*	ZDM 0083	1510	1190	320	3.72	[32]
*Daxiatitan binglingi*	FRDC-GSLTZP03-001	2040	1680	460	3.65	[14]
*Alamosaurus sanjuanensis*	USNM 15560	1948	1475	473	3.12	[49]
*Shunosaurus lii*	T5402	1232	902	330	2.73	[5]
*Euhelopus zdanskyi*	unnumbered (“exemplar C”)	1512	1200	460	2.61	[2]
*Huanghetitan liujiaxiaensis*	GSLTZP02-001	1730	1230	500	2.46	[14]
*Diplodocus carnegii*	CM 84	1600	1240	512	2.42	[45]
*Opisthocoelicaudia skarzynskii*	ZPAL MgD-I/25c	1700	1140	480	2.38	[53]
*Camarasaurus supremus*	AMNH 5761/sc.1	2390	1665	725	2.30	[44]

Notes: L(SC) refers to the maximum length of the scapulocoracoid; L(S) refers to the maximum length of the scapula; L(C) refers to the maximum length of the coracoid; SCI is the index of the maximum length of the scapula divided by that of the coracoid. Abbreviations: AMNH, American Museum of Natural History, New York, U.S.A.; CM, Carnegie Museum of Natural History, Pittsburgh, PA, U.S.A.; FRDC, Fossil Research and Development Center, Lanzhou, China; USNM, United States National Museum (now NMNH, National Museum of Natural History; Smithsonian Institution) Washington, DC, U.S.A.; ZDM, Zigong Dinosaur Museum, Zigong, China; ZPAL, Institute of Paleobiology, Polish Academy of Sciences, Warsaw.

In addition to the scapula, the coracoid presents some distinct features. It resembles the general morphology of the coracoid of *Opisthocoelicaudia*, but its cranioventral corner is rounder; it is not as square as in *Opisthocoelicaudia* and *Alamosaurus*. Although there still appears to be a slight gap between the scapula and coracoid–the cranial margin of the coracoid does not extend as far as that of the scapula, and the cranial margins of coracoid and scapula were missing part of structure at the synostosis–they still tend to merge smoothly, as in *Opisthocoelicaudia* but unlike the strikingly V-shaped, re-entrant cranial margins in *Daxiatitan*, *Alamosaurus* and *Euhelopus* (Fig. 16). The position of the coracoid foramen in *Yongjinglong* is the most unusual feature in the scapulocoracoid. In reptiles in general, the coracoid foramen allows passage for the supracoracoid nerve and artery from the axilla to the lateral side of the shoulder [27]. In most sauropods, the coracoid foramen is teardrop-shaped and located entirely within the coracoid. Typically, the coracoid foramen on the medial side lies close to the scapula–coracoid contact, but on the lateral side it is farther (more ventral) from the contact and wholly on the coracoid. The coracoid foramen of *Yongjinglong* is not fully enclosed by the coracoid; its course parallels the long axis of the scapula, but on the medial side it is uniquely almost entirely on the scapula rather than on the coracoid. Other sauropods (e.g., *Daxiatitan*, *Chubutisaurus*, *Camarasaurus*, *Saltasaurus*, and *Supersaurus* (BYU 9025)) have more typically positioned coracoid foramina. The coracoid foramen of *Tienshanosaurus* (Fig. 16) [57] is rounded and is far away from the contact on the lateral side but is triangular and adjacent to the contact on the medial side. The coracoid foramen of *Euhelopus* is closer to the craniodorsal margin of the contact on the medial side, which hangs over the ventral part of the contact. In general among Asian sauropods, the coracoid foramen on the medial side is located closer to the scapula–coracoid contact than those in North and South American sauropods. Additionally, the coracoid foramen of *Yongjinglong* is notch-shaped, which is commonly seen in *Apatosaurus* juveniles [25] (personal observation of specimens at BYU), suggesting the specimen pertains to a subadult individual.

### Ulna and Radius

The ulna and radius are shorter than those of *Opisthocoelicaudia* and *Barosaurus*, but close in length to those of smaller specimens of *Mamenchisaurus*, *Camarasaurus*, *Diplodocus* and *Apatosaurus*, which show a wide ontogenetic size range. Despite this, the lengths of large specimens of these taxa exceed those of *Yongjinglong* (Table 4) [25]. The ulna of *Yongjinglong* bears a more robust shaft with a larger least circumference than those of many other sauropods such as *Apatosaurus* and *Camarasaurus*. The angle of the triangular proximal end of the ulna is similar to that of *Camarasaurus*. Unlike other sauropods, the radius bears deep caudal and lateral fossae and grooves on the distal part that indicates the attachments of muscles and connective tissue associated with the ulna and adjacent elements. The radius robustness index is 2.7, slightly less than the value for *Opisthocoelicaudia* [53]. The proximal and distal ends are almost equally expanded, and are more than three times the minimum width of the diaphysis.

**Table 4:** Measurements (in mm) of the right ulna and radius preserved in the holotype of *Yongjinglong datangi* (GSGM ZH(08)-04.

Ulna	Size	Radius	Size
Total length	590	Total length	550
Proximal mediolateral width	340	Proximal mediolateral width	165
Craniocaudal length of the caudal process	200	Proximal craniocaudal width	165
Maximum length of craniomedial process	230	Proximal length to midshaft	260
Width of craniomedial process	75	Craniocaudal min width of shaft	60
Maximum length of craniolateral process	160	Mediolateral min width of shaft	80
Width of craniolateral process	90	Craniocaudal width of midshaft	70
Proximal length to min the shaft	450	Mediolateral width of midshaft	90
Craniocaudal min width of the shaft	60	Circumference of the midshaft	240
Mediolateral min width of the shaft	105	Distal craniocaudal width	190
Min circumference of the shaft	300	Distal mediolateral width	160
Distal mediolateral width	145		

Notes: Maximum length of craniomedial/craniolateral process refers to the distance from the tip of the caudal process to the end of the craniomedial/craniolateral process). Min = minimum.

### Skeletal Reconstruction (Fig. 17)

The shape and remarkable length of the scapula in *Yongjinglong* raise an interesting issue about its orientation relative to the dorsal axial column. Because pectoral girdle and limb elements are not solidly attached to the rest of the skeleton, they displace readily taphonomically from their *in vivo* positions. Historically, two orientations have been used for the scapula in skeletal reconstructions: a mammal-style, nearly vertical orientation [28], [58] and a subhorizontal one [25], [59]. The best preserved evidence of the more horizontal orientation is the articulated juvenile *Camarasaurus* skeleton, CM 11338. A vertical orientation results in a caudal projection of the glenoid, which is not favorable for the arc of movement of the humerus [31]. Thus, a subhorizontal position for scapula, which allows freer movement of the shoulder has gradually been accepted [60]. The exceptional length of the element in *Yongjinglong* makes finding the optimal orientation difficult. Each of the five preserved dorsal vertebral centra of *Yongjinglong* is shorter than 250 mm; if that length is presumed to be average for all dorsal centra, and assuming that *Yongjinglong* had the typical number of 12 or 13 dorsal vertebrae seen in most sauropods [29], the dorsal column would have spanned only 2500–2900 mm. The distal end of a horizontal, 1550 mm-long (straight length) scapula would have reached the seventh or eighth dorsal rib, more than half the length of the trunk, rather than the fourth to the sixth rib as in typical sauropods reconstructions [29], [30] (L. Li, in preparation). In order to restrict the scapula to a span of six or fewer ribs, the trunk of *Yongjinglong* would have to be exceptionally long either in terms of its number of dorsal vertebrae or in the lengths of the many of unpreserved dorsal vertebrae in the holotype. In a vertical orientation, the scapular blade would span only few ribs, but it would add unreasonably to the height of the shoulder. A subvertical (approximately 50° from horizontal) scapular orientation (Fig. 17) puts the dorsal end of the scapula in the middle trunk region of *Yongjinglong*. This arrangement provisionally suggests how the very long scapula may be accommodated on the short trunk.

### Phylogenetic Analysis

To assess the phylogenetic position of *Yongjinglong datangi*, we used the data matrices of Mannion et al. [18] and D’Emic [19] as well as that of Carballido & Sander [61] (see Table S1 (Mannion et al.), Table S2 (D’Emic) and Table S3 (Carballido & Sander 2013)). The matrix of Carballido & Sander comprised 71 sauropods and 341 characters; that of D’Emic, which emphasized early branching titanosauriforms, comprised 26 sauropods and 119 characters. The matrices of Mannion et al. comprised 64 taxa, including nine Chinese sauropods that had never previously been evaluated in a phylogenetic analysis. Furthermore, Mannion et al. ran two separate analyses. The first one (LCDM), with 279 characters, included 74 continuous characters, whereas the other one (LSDM) had the same characters but regarded the 74 characters as discrete. We used the LSDM to retrieve the phylogenetic position of *Yongjinglong.* We postpruned 11 operational taxonomic units (*Australodocus*, *Daxiatitan*, *Fukuititan*, *Fusuisaurus*, *Ligabuesaurus*, *Lusotitan*, *Malarguesaurus, “Pelorosaurus”_becklesii, Tastavinsaurus*, *Tendaguria*, and *Wintonotitan*) due to their unstable positions. The preserved elements in the holotype of *Yongjinglong datangi* allowed coding of 74 characters in the selected matrix of Mannion et al. (Fig. 18A). The matrices of D’Emic and Carballido & Sander allowed the coding of 34 and 58 characters, respectively (Fig. 18B, C). The Mannion et al. matrix was run in TNT, using a New Technology search [62], and it yielded 32878 most parsimonious trees (MPTs) of length 1063 steps (consistency index (CI) = 0.278; retention index (RI) = 0.543); the D’Emic matrix was run in TNT, using a traditional search; it recovered two MPTs of 208 steps (CI = 0.601; RI = 0.801). The matrix of Carballido & Sander [61] was run in TNT using an equally weighted parsimony analysis; a heuristic tree search was performed starting from 1000 replicates of Wagner trees (with random addition sequence of taxa) followed by TBR branch swapping (saving 10 trees per replicate). This procedure retrieved eight most parsimonious trees (MPTs) of 1018 steps (CI = 0.398; RI = 0.720), found in 437 of the replicates. In order to keep the taxon set congruent with our other analyses, we pruned 31 taxa in our analysis, most of which were non-Titanosauriformes but also including *Daxiatitan*, pending a more detailed description and analysis of that taxon [L. Li in preparation]. Ultimately, 40 taxa were retained. The three different analyses agree in recovering *Yongjinglong datangi* as strongly derived within Somphospondyli among more derived Titanosauriformes. Specifically, based on the D’Emic and Carballido & Sander analyses, *Yongjinglong* was recovered within Titanosauria as the sister taxon to *Opisthocoelicaudia*, adjacent to *Alamosaurus* (Fig. 18B, C). In the Mannion et al. matrix, *Yongjinglong* was recovered as the sister taxon of the Australian sauropod *Diamantinasaurus* (Fig. 18A). A constrained analysis with the Carballido & Sander matrix forced *Yongjinglong* into a position as sister taxon to *Phuwiangosaurus*; this would make *Yongjinglong* part of the endemic, Asian Euhelopodidae recovered by the analysis of D’Emic ([19], figs. 5, 6). Four extra steps are required for this shift in position. Characters 230 and 231 are the synapomorphies that unite *Opisthocoelicaudia* and *Yongjinglong* in the Carballido & Sander [61] analysis. Five characters (149, 165, 236, 242 and 243) would unite *Yongjinglong* with *Phuwiangosaurus*. However, all three analyses recovered *Yongjinglong datangi* as a member of Somphospondyli, and two recovered it as a sister taxon of the Asian titanosaurian *Opisthocoelicaudia*. *Yongjinglong* is thus deeply nested within Titanosauriformes and recovered as one of the most derived titanosaurian sauropods in Asia.

## Discussion and Conclusions

*Yongjinglong datangi* is a new titanosaurian sauropod from the Lower Cretaceous Hekou Group of Gansu Province, northwestern China, a unit that has yielded abundant fossils in the last decade. The new species exhibits several autapomorphies and unique combinations of characters: (1) long-crowned spoon-shaped premaxillary teeth; (2) axially elongate parapophyses in the caudal cervical vertebra; (3) deep, undivided pneumatic foramina on the lateral surfaces of the cervical vertebra; (4) an “XI” (or “IX”)-shaped configuration of laminae on the left (or the right) lateral surfaces of the middle dorsal vertebrae; (5) a low, unbifurcated neural spine with the postzygapophyses forming a cranially-pointing triangular plate in the middle dorsal vertebrae; (6) very long scapula with straight, parallel edges of the scapular blade; and (7) a tall, deep groove embaying the lateral surface of the distal shaft of the radius. *Yongjinglong* also shares several synapomorphies with other sauropods: a pronounced *M. triceps longus* tubercle caudoventral to the acromial edge of the scapular plate as in *Chubutisaurus* and *Daxiatitan*; and ventrally elongate parapophyses in its cervical vertebra, as in *Daxiatitan* and *Euhelopus*.

The notch-like coracoid foramen enclosed medially by the scapula and the partially fused scapulocoracoid suggests that the holotype specimen may pertain to a subadult individual. The remarkably long scapulocoracoid presents a challenge for understanding the correct orientation of the scapulocoracoid. Considering the orientation of glenoid and the extension of the distal border of the scapula, a subvertical orientation requires an unlikely elevation of the shoulder, while a subhorizontal orientation requires an unusually long extension of the distal border of the scapula across too many ribs. In the latter case, either there were several hyper-elongate missing dorsal vertebrae are not preserved in the holotype, or else there was an exceptionally large number of missing dorsal vertebrae. However, because no well-preserved sauropod specimen shows dramatic changes in the sizes or the number of dorsal vertebrae, the more probable solution is a modest 50° elevation of the scapula above horizontal, somewhat less than the 55–65° angles proposed for other sauropods by Schwartz et al. [60].

Titanosauriformes were not recognized in China until 1998 when *Euhelopus*, the first sauropod reported from China, was reinterpreted [63]. Subsequently, confirmation of Titanosauriformes as a major component of Chinese faunas came with the descriptions of *Gobititan* in 2003, *Huanghetitan liujiaxiaensis* in 2006, and *Daxiatitan* in 2008. Titanosauriformes constitute 30% of the sauropods so far reported from China (L. Li, in preparation), and 100% of Cretaceous Chinese sauropods are Titanosauriformes [33]. In this study, *Yongjinglong* is recovered as a derived titanosauriform in all three phylogenetic analyses, especially in the matrices of Carballido & Sander [61] and D’Emic [19], in which it occupies a position within Titanosauria. It is currently difficult to compare *Yongjinglong* with *Erketu ellisoni* because few elements overlap between available specimens. However, *Yongjinglong* shares some characters with *Euhelopus* in Euhelopodidae and *Opisthocoelicaudia* in Titanosauria. *Yongjinglong* exhibits several features similar to *Euhelopus*, but the phylogenetic results show a wide separation between the two taxa within Somphospondyli. *Euhelopus* is recovered in a basal position relative to *Yongjinglong* within Somphospondyli, either in a monophyletic Euhelopodidae, as with the matrices of Mannion et al. [18] and D’Emic [19], or by itself, as with the matrix of Carballido & Sander [61]. These results mean that the apparent similarities in dorsal vertebral laminae in *Yongjinglong* and *Euhelopus* evolved independently.

*Yongjinglong datangi* is the third genus of sauropod from the Hekou Group. *Euhelopus* comes from Shandong Province, in northeastern China; the fossil localities of *Yongjinglong*, *Daxiatitan*, and *Huanghetitan liujiaxiaensis* in northwestern China are very close to each other. The three Hekou Group sauropods were members of the same fauna, but each is distinct and has numerous autapomorphies. *Yongjinglong* shows more similarities with *Euhelopus* in its vertebrae, but is distinct from *Euhelopus* in its appendicular skeleton, which more closely resembles that of *Opisthocoelicaudia*. *Yongjinglong datangi* will play a key role for investigating the relationships of *Euhelopus*, *Opisthocoelicaudia*, and other Hekou group Titanosauriformes in future research. The phylogenetic results have implications for sauropod paleobiogeographic research: a position for*Yongjinglong datangi* within Titanosauria may elucidate the origin and radiation of the different groups of Titanosauriformes in Asia and other continents during the Early Cretaceous. The present specimen emphasizes the high diversity of Early Cretaceous Titanosauriformes in China and affirms that China was a center of diversity of Titanosauriformes at this time.

## Supporting Information

Table S1 **Character coding for** ***Yongjinglong datangi*** **gen. et sp. nov. based on the matrix of Mannion et al. (2013).** The Mannion et al. matrix LSDM, with 279 characters, included 74 continuous characters as discrete. The preserved elements in the holotype of *Yongjinglong datangi* allowed coding of 74 characters.
(TNT)

Table S2 **Character coding for** ***Yongjinglong datangi*** **gen. et sp. nov. based on the matrix of D’Emic (2012).** The matrix of D’Emic allowed the coding of 34.
(TNT)

Table S3 **Character coding for** ***Yongjinglong datangi*** **gen. et sp. nov. based on the matrix of Carballido & Sander (2013).** The matrix of Carballido & Sander allowed the coding of 58 characters.
(TNT)
